# Genome-wide mRNA expression profiling in *vastus lateralis* of COPD patients with low and normal fat free mass index and healthy controls

**DOI:** 10.1186/s12931-014-0139-5

**Published:** 2015-01-08

**Authors:** Roberto A Rabinovich, Ellen Drost, Jonathan R Manning, Donald R Dunbar, MaCarmen Díaz-Ramos, Ramzi Lakhdar, Ricardo Bastos, William MacNee

**Affiliations:** ELEGI Colt Laboratory, Centre for Inflammation Research, The Queen’s Medical Research Institute, University of Edinburgh, 47 Little France Crescent, Edinburgh, Scotland EH16 4TJ UK; Centre for Cardiovascular Science, University of Edinburgh, Scotland, UK; Institut d’Investigacions Biomèdiques August Pi i Sunyer (IDIBAPS), Barcelona, Spain; Ciber de Enfermedades Respiratorias (CIBERES), Barcelona, Spain

**Keywords:** COPD, Skeletal Muscle Dysfunction, Skeletal muscle wasting, Gene expression, Ageing

## Abstract

**Background:**

Chronic Obstructive Pulmonary Disease (COPD) has significant systemic effects beyond the lungs amongst which muscle wasting is a prominent contributor to exercise limitation and an independent predictor of morbidity and mortality. The molecular mechanisms leading to skeletal muscle dysfunction/wasting are not fully understood and are likely to be multi-factorial. The need to develop therapeutic strategies aimed at improving skeletal muscle dysfunction/wasting requires a better understanding of the molecular mechanisms responsible for these abnormalities. Microarrays are powerful tools that allow the investigation of the expression of thousands of genes, virtually the whole genome, simultaneously. We aim at identifying genes and molecular pathways involved in skeletal muscle wasting in COPD.

**Methods:**

We assessed and compared the *vastus lateralis* transcriptome of COPD patients with low fat free mass index (FFMI) as a surrogate of muscle mass (COPD_L_) (FEV_1_ 30 ± 3.6%pred, FFMI 15 ± 0.2 Kg.m^−2^) with patients with COPD and normal FFMI (COPD_N_) (FEV_1_ 44 ± 5.8%pred, FFMI 19 ± 0.5 Kg.m^−2^) and a group of age and sex matched healthy controls (C) (FEV_1_ 95 ± 3.9%pred, FFMI 20 ± 0.8 Kg.m^−2^) using Agilent Human Whole Genome 4x44K microarrays. The altered expression of several of these genes was confirmed by real time TaqMan PCR. Protein levels of P21 were assessed by immunoblotting.

**Results:**

A subset of 42 genes was differentially expressed in COPD_L_ in comparison to both COPD_N_ and C (PFP < 0.05; −1.5 ≥ FC ≥ 1.5). The altered expression of several of these genes was confirmed by real time TaqMan PCR and correlated with different functional and structural muscle parameters. Five of these genes (CDKN1A, GADD45A, PMP22, BEX2, CGREF1, CYR61), were associated with cell cycle arrest and growth regulation and had been previously identified in studies relating muscle wasting and ageing. Protein levels of CDKN1A, a recognized marker of premature ageing/cell cycle arrest, were also found to be increased in COPD_L_.

**Conclusions:**

This study provides evidence of differentially expressed genes in peripheral muscle in COPD patients corresponding to relevant biological processes associated with skeletal muscle wasting and provides potential targets for future therapeutic interventions to prevent loss of muscle function and mass in COPD.

**Electronic supplementary material:**

The online version of this article (doi:10.1186/s12931-014-0139-5) contains supplementary material, which is available to authorized users.

## Introduction

Chronic obstructive pulmonary disease (COPD) is associated with several extra-pulmonary effects of which skeletal muscle wasting is one of the most extensively studied [1,2] and results in loss of muscle strength [1,3-6], contributes to exercise (in)tolerance [7-10] and is a predictor of health related quality of life (HRQoL) [11] and survival [12,13] independent of the degree of airway obstruction [10].

Muscle wasting affects 18 to 36% of patients with COPD [7,14] and can be present even in patients with normal weight [7,14,15]. Indeed, muscle wasting is a better predictor of health related quality of life [11] and survival [12,13] than body weight itself. But why does only a subgroup of patients with COPD develop muscle wasting?; Several patho-physiological changes, have been identified in the skeletal muscle of COPD patients namely fibre size reduction (atrophy) [7], fibre type redistribution [16], altered bioenergetics [16], altered capillarization [17], and altered mitochondrial function [18,19].

The molecular mechanisms leading to skeletal muscle wasting are not fully understood and are likely to be multi-factorial, including physical inactivity, systemic inflammation/oxidative stress and cell hypoxia [1] among others.

Accumulating evidence supports the idea that COPD is a disease of accelerated ageing [20]. It has recently been shown that limb muscles of patients with COPD have increased number of senescent satellite cells and an exhausted muscle regenerative capacity, compromising the maintenance of muscle mass in these individuals [21] suggesting that premature cellular senescence and subsequent exhaustion of the regenerative potential of the muscles may relate to muscle abnormalities characteristic of these patients.

Strategies to reverse skeletal muscle dysfunction/wasting achieve only relatively modest improvements [22]. There is a need to develop therapeutic strategies aimed at improving skeletal muscle dysfunction/wasting, which requires a better understanding of the molecular mechanisms responsible for these abnormalities.

Microarrays are powerful tools that allow the investigation of the expression of thousands of genes, virtually the whole genome, simultaneously. An analysis of the genes that are being transcribed in the muscle, the transcriptome, should shed light on the molecular mechanisms responsible for muscle dysfunction and wasting in COPD and can help to identify molecular targets for the development of therapeutic strategies specifically designed to improve muscle function and bulk.

In this study we assessed the transcriptome of the *vastus lateralis* muscle in COPD patients with low fat free mass index (FFMI) as a surrogate of muscle mass (COPD_L_) in comparison to patients with COPD and normal FFMI (COPD_N_) and a group of age and sex matched healthy controls (C).

We hypothesize that genes related to cell cycle arrest and inhibition of cell growth will be up-regulated while genes related to energy production and muscle development will be down-regulated in COPD_L_. We expect similarities in the transcriptome of COPD_L_ and muscle wasting relating to the normal ageing process. Moreover, the transcriptome analysis of this group may reveal important pathways leading to peripheral muscle wasting.

This study demonstrates that *vastus lateralis* of patients with COPD and muscle wasting overexpress genes related to inhibition of cell cycle and of cell growth whilst genes related to muscle formation and growth and energy production were down-regulated. This pattern is similar to observations associated with ageing, which suggests that premature ageing may play a role in muscle atrophy in COPD.

## Methods

### Study group

Nineteen stable patients with COPD, nine with low FFMI (COPD_L_) and ten with normal FFMI (COPD_N_), and ten age, gender and smoking status-matched healthy subjects with normal FFMI were included in the present study (Table 1). All patients had a diagnosis of COPD according to the Global Initiative for Chronic Obstructive Lung Disease [23]. They were clinically stable and free of exacerbations for 4 weeks prior to the study and free of drugs that can potentially affect the muscle (i.e. systemic corticosteroids, statins). The study was approved by the Lothian Regional Ethics Committee.

**Table 1:** **Characteristics of the study groups**

	**COPD**_**L**_		**COPD**_**N**_		**Controls**		**p-value**
M/F	7/2	**A**	8/2	**A**	8/2	**A**	ns
Age (Years)	67 ± 2.0	**A**	69 ± 1.5	**A**	68 ± 1.4	**A**	ns
BMI (Kg.m^−2^)	18.8 ± 0.7	**A**	26 ± 0.7	**B**	30 ± 2.1	**C**	**<0.0001**
FFMI (Kg.m^−2^)	15 ± 0.2	**A**	19 ± 0.5	**B**	20 ± 0.8	**B**	**<0.0001**
Active/ex-smokers	1/8	**A**	2/8	**A**	2/8	**A**	ns
Pack/year	66 ± 14	**A**	49 ± 6.6	**AB**	32 ± 5.0	**B**	**0.037**
Average cessation (years)	6.0 ± 2.5	**A**	8.4 ± 2.2	**A**	23 ± 5.5	**B**	**<0.01**
Age at smoking cessation (years)	61.1 ± 8.2	**A**	60.5 ± 7.6	**A**	45.1 ± 15.5	**B**	**<0.01**
mMRC	4 ± 0.4	**A**	3 ± 0.4	**B**			**0.038**
FEV_1_ (L)	0.8 ± 0.1	**A**	1.2 ± 0.1	**A**	2.8 ± 0.2	**B**	**<0.0001**
FEV_1_ (% pred)	30 ± 3.6	**A**	44 ± 5.8	**A**	95 ± 3.9	**B**	**<0.0001**
FVC (L)	2.6 ± 0.3	**A**	2.7 ± 0.4	**A**	3.9 ± 0.2	**B**	**0.015**
FVC (% pred)	76 ± 6.4	**A**	88 ± 9.0	**AB**	104 ± 3.2	**B**	**0.01**
FEV_1_/FVC	0.32 ± 0.1	**A**	0.38 ± 0.1	**A**	0.71 ± 0.0	**B**	**<0.0001**
PaO_2_ (mmHg)	79 ± 7.4	**A**	70 ± 3.1	**A**	73 ± 1.8	**A**	ns
PaCO_2_ (mmHg)	43 ± 2.0	**A**	41 ± 1.1	**A**	42 ± 0.6	**A**	ns
Physical activity (V)	1.2 ± 0.3	**A**	7 ± 1.9	**B**	11 ± 1.5	**B**	**<0.0001**
Physical activity (L)	43 ± 3.6	**A**	30 ± 5.0	**A**			ns
QMVC (N)	213 ± 19.4	**A**	323 ± 25.6	**B**	366 ± 26.8	**B**	**0.002**
6MWD (m)	306 ± 55	**A**	411 ± 50.1	**AB**	524 ± 37.9	**B**	**0.01**
BODE	6.5 ± 1.3	**A**	4 ± 0.9	**A**			ns
SGRQ Symptoms	76 ± 4.8	**A**	60 ± 4.2	**B**			**0.01**
SGRQ Activity	86 ± 4.7	**A**	52 ± 9.2	**B**			**0.003**
SGRQ Impact	57 ± 6.6	**A**	33 ± 7.7	**B**			**0.01**
SGRQ Total	69 ± 5.4	**A**	43 ± 6.8	**B**			**0.005**
Type I Fibre (%)	25.1 ± 4.6	**A**	24.4 ± 3.3	**A**	38.7 ± 3.9	**B**	**0.04**
Type II area (μ^2^)	2033 ± 166	**A**	2978 ± 277	**B**	2564 ± 277	**AB**	**0.03**

*Definition of abbreviations*: COPD_N_ = COPD patients with normal FFMI; COPD_L_ = patients with COPD with low FFMI; BMI = Body mass index; FFMI = fat free mass index; MRC = medical research council dyspnoea score; FEV_1_ = forced expiratory volume in the first second; FVC = forced vital capacity; PaO_2_ = arterial oxygen partial pressure; PaCO_2_ = arterial carbon dioxide partial pressure; Physical Activity (V) = Voorrips Questionnaire; Physical activity (L) = London Chest Activity of Daily Living Scale; QMVC = quadriceps maximal voluntary contraction; 6MWD = six minute walking distance; SGRQ = St. George’s Respiratory Questionnaire; ns = not significant; NA = not applicable. Comparisons among groups were done using ANOVA and Student-Newman-Keuls as a post-hoc test. Differences among the three different groups were stated using letters A, B and C where sharing a letter implies no differences between these groups and having a different letter implies a statistical difference in the post-hoc test.

### Measurements

#### Assessment

All subjects had the following baseline assessments: anthropometric measurements, body composition measurement with bioimpedance (BIA), pulmonary function tests (spirometry) and blood gases (Ciba Corning 800, USA), six–minute walking distance (6MWD) [24], quadriceps maximal voluntary contraction (QMVC) [25] (Chatillon® K-MSC 500, Ametek, Florida), health-related quality of life questionnaires (St. George’s Respiratory Questionnaire, SGRQ) [26], modified Medical Research Council (mMRC) dyspnoea scale and physical activity (PA) levels using the Voorrips physical activity questionnaire (PA_V_) [27] and the London Chest Activity of Daily Living Scale (LCADL) [28] for patients with COPD. Number of exacerbations in the previous year was recorded.

Low FFMI was defined as <16 kg.m^−2^ for male and <15 kg.m^−2^ for female COPD patients [29].

### *Vastus Lateralis* muscle biopsy and RNA isolation

An open muscle biopsy of the *“vastus lateralis”* was obtained and ~0.1 g was included in RNA stabilization reagent (RNAlater®, Ambion, Inc., USA) and stored at −20°C for RNA extraction. Total RNA was extracted and purified by homogenisation (TissueLyser, Qiagen Ltd. West Sussex, UK) of tissue employing the TRIzol® Plus RNA Purification Kit (Invitrogen Life Technologies, Carlsbad, CA).

### Fibre type typification

Paraffin sections (5um) were de-waxed and re-hydrated through graded ethanol using standard procedures. Sections were placed in 250 ml of Novocastra pH8 retrieval buffer and subjected to antigen retrieval in a de-cloaking chamber (Biocare Medical, USA) using a protocol described elsewhere [30].

### Microarray hybridization and data analysis

Five hundred nanograms of total RNA from each sample was converted into labelled cRNA with nucleotides coupled to a fluorescent dye (Cy3) using the Quick Amp Kit (Agilent Technologies, Palo Alto, CA). Cy3-labeled cRNA (1.65 μg) was hybridized to Agilent Human Whole Genome 4×44K Microarrays (Agilent Technologies, Santa Clara, CA). The hybridized array was then washed and scanned and the data were extracted from the scanned image using Feature Extraction version 10.2 (Agilent Technologies).

Pre-processing (background correction, normalization, filtering and summarization) subsequent data processing and analysis was performed using the Agi4x44 Pre-process module from *Bioconductor* [31,32].

The Rank Product [33,34] was employed for the microarray data analysis. RP is a non-parametric algorithm that detects probes/genes consistently highly ranked by fold-change between samples from different groups and employs a ‘percent false positives’ (PFP) measure, also known as false discovery rate (FDR), to select the most significant differential expressions. A percentage of false positive (PFP) below 0.05 was considered statistically significant.

The gene functional enrichment analysis was performed using DAVID Bioinformatics Resources (http://david.abcc.ncifcrf.gov/). Specifically, the Functional Annotation Chart tool was used to enrich the over-represented Gene Ontology (GO) terms among the differentially expressed gene list. A list of all detectable transcripts was used as the background for the GO analysis [35]. The GO terms after correction for FDR at p < 0.05 (Benjamini Hochberg) were selected for further analysis and interpretation.

Ingenuity Pathway Analysis (IPA, Ingenuity Systems, Redwood, CA) was used to further investigate the Genespring expression clusters [36].

### qPCR validation

Based on microarray-derived fold-change (>2) or statistical significance for differential expression and/or the biological relevance for the different comparisons, 11 genes were selected for TaqMan qPCR validation (Applied Biosystems).

### Western blot analysis

P21, the protein encoded by the CDKN1A gene was determined using immunoblotting. 20 μg of protein was resolved by sodium-dodecyl sulfate-polyacrylamide gel electrophoresis on 10% polyacrylamide gels. Proteins were transferred to Immobilon-P PVDF membranes (Millipore, Billerica, MA), blocked with 5% dry milk (Bio-Rad, München, Germany) in TBS (Sigma) overnight at 4°C and probed with primary antibodies primary antibody against p21 (ab 7960) (Abcam, Bristol, UK) during 1 h at room temperature. Proteins were then visualized using the ECL Detection System (Pierce, Rockford, IL) as per the manufacturer’s instructions.

### Statistical analysis

Anthropometric, physiological data and immunobloting results for p21 are expressed as mean ± SEM. These data were analysed using ANOVA with Student-Newman-Keuls as a post-hoc test. Correlation analysis between variables was conducted using Pearson’s correlation index for continuous variables and Spearman’s correlation index for categorical variables. For the qPCR validation analysis differential expression analysis on individual sample values of ΔCT using Kruskal Wallis with a Nemenyi-Damico-Wolfe-Dunn post-hoc test was performed.

The statistics were conducted using the statistical package SAS version 9.3 (SAS Institute Inc, Cary, NC, USA). A p value <0.05 was taken as statistically significant.

Full details of the methods can be found in the Additional file 1.

## Results

Anthropometric characteristics and pulmonary function data of study subjects are depicted in Table 1. Both groups of COPD patients showed airflow limitation compared to healthy controls (C) who all had normal spirometry, but there were no differences in spirometry between COPD_N_ and COPD_L_ (Table 1 and Additional file 2: Figure S1). Patients were matched by smoking status (two COPD_N_, one COPD_L_ patient and two healthy controls were active smokers at the time of inclusion, p = ns). All subjects, including healthy controls, were exposed (current or ex-smokers) to cigarette smoking (no never-smokers were included in the study). Moreover, no differences in the number of years of smoking cessation was seen between COPD_L_ (6.0 ± 2.5 years) and COPD_N_ (8.4 ± 2.2 years) nor in the age of the patients when they stopped smoking (61.1 ± 8.2 years old) COPD_L_ and (60.5 ± 7.6 years) COPD_N_. Both COPD groups showed less years of smoking cessation than controls (23.2 ± 5.5 years) (ANOVA P < 0.01).

Compared to C, COPD_N_ had lower BMI and physical activity and COPD_L_ had very different anthropometric characteristics.

Compared to COPD_N_, COPD_L_ had significantly lower BMI, FFM and FFMI, poorer HRQoL with higher values in all of the domains of the St. George’s respiratory questionnaire, higher mMRC score and worse muscle function as assessed by QMVC. They also had lower levels of physical activity measured by the Voorrips questionnaire (PA_V_) but no difference were seen in activities of daily living (ADL) assessed with the LCADL (PA_L_), specifically designed to assess ADL in patients with COPD (Table 1).

Both COPD groups showed a redistribution of muscle fibre type with a higher proportion of type II fibres and a lower proportion type I in comparison to healthy controls. Type II fibre area was significantly reduced in COPD_L_ in comparison with COPD_N_ (Table 1).

In the whole COPD population FFMI correlated with FEV_1_ (r = 0.51, p < 0.05), mMRC dyspnoea score (rho = −0.48, p < 0.05), QMVC (r = 0.76, p < 0.001), and physical activity (PA_V_) (r = 0.61, p < 0.01) while skeletal muscle function (QMVC) correlated with FFMI (r = 0.76, p < 0.0001), exacerbation rate (rho = −0.57, p < 0.05), 6MWD (r = 0.62, p < 0.01), physical activity (PA_V_) (r = 0.53, p < 0.05) and BODE index (rho = −0.66, p < 0.05).

### Global assessment of gene expression

Hierarchical and k-means clustering were undertaken with normalized data. No pattern emerged from this analysis. We therefore performed three pair-wise class comparisons: COPD_L_*vs*. COPD_N,_ COPD_L_*vs*. C and COPD_N_*vs*. C, employing Rank Products (RP) to detect differentially expressed genes (DEGs). First, a list of up- or down-regulated genes for each comparison was selected based on a PFP <0.05 (Table 2) (no FC criterion was applied). Comparisons of both groups COPD patients with C showed the most differentially expressed genes and the comparison between the COPD groups the least.

**Table 2:** **DEGs between COPD**_L_ **, COPD**_N_ **and C**

		**COPD**_**L**_ **vs COPD**_**N**_	**COPD**_**L**_ **vs C**	**COPD**_**N**_ **vs C**
**Up regulated**	Probes	954	1352	1050
	Genes	531	830	724
**Down regulated**	Probes	1054	1631	1201
	Genes	748	1152	799

DEGs between COPD_L_ vs. COPD_N_, COPD_L_ vs. C and COPD_N_ vs C. Number of up and down regulated probes and genes with a PFP < 0.05 in all three comparisons.

**Figure 1:**
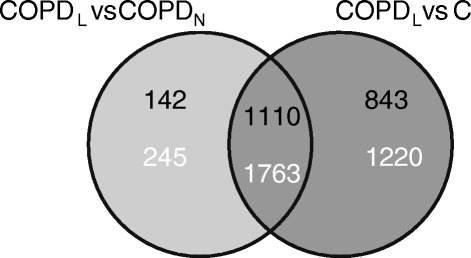
**Venn diagram showing the numbers of genes (black) and probes (white) differentially expressed between COPD**_L_ **and both other groups without muscle wasting (COPD**_N_ **and C) (PFP < 0.05).**

In order to select the most relevant DEGs related to muscle wasting in COPD, we selected a list of DEGs between COPD_L_ and COPD_N_. Furthermore, among this list, we selected those genes that were also differentially expressed between COPD_L_ and C (Figure 1). This list of 1110 DEGs (1763 probes) (454 DEGs up-regulated and 656 DEGs down-regulated) was used to conduct a functional enrichment analysis. Table 3 displays the most characteristic GO biological processes (with 8 or more genes representing each class/category/pathway) that were enriched in this list. Significant GO classes from the list of up-regulated DEG correspond to functional terms related to protein synthesis, muscle organ development and striated muscle contraction while functional terms related to glucose metabolism, energy production, striated muscle development and striated muscle contraction were identified from the list of down-regulated genes.

**Table 3:** **GO Terms associated with relevant genes related to muscle wasting**

**Source**	**GO terms**	**Fold enrichment**	**N** ^**o**^ **of gene in the term**	**FDR**
**Up-regulated genes**	Traslational initiation	7.9	11	0.001
	Traslational elongation	14.7	49	6.05E-42
	Ribosomal biogenesis	4.2	17	0.004
	rRNA processing	4.9	15	0.002
	Muscle organ development	3.2	20	1.62E-05
	Striated muscle contraction	6.5	10	1.55E-05
**Down-regulated genes**	Glucose metabolic process	3.3	21	0.008
	Energy derivation by oxidation of organic compounds	5.8	37	4.86E-15
	Electron transport chain	6.2	31	5.77E-13
	ATP synthesis coupled electron transport	9.3	22	1.91E-12
	Striated muscle tissue development	3.4	18	0.02
	Muscle contraction	4.7	31	1.94E-09

GO Terms associated with DEGs between COPD_L_ vs. COPD_N_ and COPD_L_ vs. C.

Functional enrichment analysis of 454 up-regulated and 656 down-regulated DEGs between COPD_L_ vs. COPD_N_ and COPD_L_ vs. C (FDR < 0.05) with the corresponding GO term, the fold enrichment, number of genes involved in the term and the FDR (False discovery Rate).

**Figure 2:**
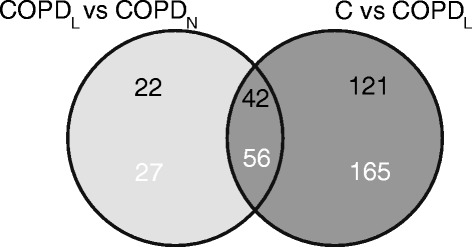
**Venn diagram showing the numbers of genes (black) and probes (white) differentially expressed between COPD**_L_ **and both other groups without muscle wasting (COPD**_N_ **and C) (PFP < 0.05; −1.5 ≥ FC ≥ 1.5).**

Since some of these DEGs displayed marginal changes in gene expression, we applied a more restrictive criterion, namely a fold change cutoff of 1.5 (1.5 ≥ FC ≥ 1.5) in order to select more robust genes. Figure 2 shows the list of DEGs generated between COPD_L_ and both other groups of subjects with normal muscle mass using this criterion. When the combined criteria of both statistical significance and fold change were applied the numbers of DEGs were notably reduced. Eighty three probes, representing 64 DEGs, were identified between COPD_L_ and COPD_N._ Fifty six of these probes, representing 42 DEGs, were also differentially expressed between COPD_L_ and C (Table 4 and 5). These 42 DEGs were selected for further analysis. Although this shorter list of genes was not suitable for a functional enrichment analysis, the biological significance of these genes was related with the significant functional categories identified in the list of 1110 DEGs originally obtained with a PFP < 0.05. Among the up-regulated genes (Table 4) from this list of 42 DEGs, we have identified genes related to cell cycle inhibition and inhibition of cell growth (CDKN1A, GADD45A, PMP22, BEX2, CGREF1, CYR61), inhibition of sarcomeric organization (HDGF), stress inducible factor (ATF3), inhibition of glucose metabolism (PDK4), lipid metabolism (LPL), aminoacid transportation (SLC22A3, SLC38A1), muscle repair (ABRA) and myogenesis (NNMT, ANKRD1). Among the down-regulated genes (Table 5) we have identified genes related to fatty acid synthesis (MCAT), muscle formation (CHRDL2, IRX4, PMEPA1), glucose endocytosis (RAB10) and gluconeogenesis (GPT).

**Table 4:** **Up-regulated DEGs between COPD**_L_ **vs. COPD**_N_ **and COPD**_L_ **vs. C with a FC over 1.5**

**PROBE**	**Gene symbol**	**COPD**_**L**_ **vs. COPD**_**N**_		**COPD**_**L**_ **vs. C**	
		**PFP**	**FC**	**PFP**	**FC**
A_32_P200144	**IGHG1**	0.044	5.3	0.0177	9.4
A_23_P43979	**IGLL5**	0.0435	5.1	0.0133	7.9
A_24_P104980	**IGLL5**	0.0252	4.3	0.0098	6.5
A_23_P398566		0.0133	2.9	0.0491	1.7
A_23_P46429	**CYR61**	0	2.8	0	2.9
A_24_P370946	**CYR61**	0.0009	2.7	0.0013	2.7
A_23_P46426	**CYR61**	0.0008	2.5	0.0005	2.6
A_23_P161218	**ANKRD1**	0.0074	2.5	0.0067	1.9
A_24_P376707	**HDGF**	0.0402	2.5	0.0169	2.6
A_23_P19733	**SLC22A3**	0.0004	2.4	0	4.6
A_23_P34915	**ATF3**	0.0062	2.1	0.0011	3.4
A_32_P60459	**OTUD1**	0	2.0	0	2.6
A_32_P219135		0.012	2.0	0.0042	2.4
A_23_P166248	**RCAN1**	0.0162	1.9	0.0417	1.6
A_23_P22735	**BEX2**	0.0085	1.9	0.0003	3.2
A_23_P127584	**NNMT**	0	1.9	0	3.0
A_23_P100711	**PMP22**	0	1.8	0	2.2
A_23_P49338	**TNFRSF12A**	0.0022	1.8	0.0019	1.8
A_23_P363399	**SLC38A1**	0.0257	1.8	0.0027	3.0
A_23_P146233	**LPL**	0.0003	1.8	0.0022	1.5
A_24_P243749	**PDK4**	0	1.7	0	1.6
A_23_P403445	**CGREF1**	0.0353	1.7	0.0446	1.6
A_24_P193295	**RAB15**	0.0004	1.7	0.0002	1.9
A_23_P208540	**LOC644482**	0.0095	1.7	0.0026	1.8
A_24_P261734	**SLC38A1**	0.0148	1.7	0.0012	2.9
A_23_P408095	**DSTN**	0.0091	1.6	0.033	1.6
A_23_P418031		0.0133	1.6	0.0169	1.7
A_23_P314024	**HLA-F**	0.0075	1.6	0.0026	1.8
A_23_P166109	**FLRT3**	0.0419	1.6	0.0145	1.8
A_23_P59210	**CDKN1A**	0.0074	1.5	0.0014	2.2
A_23_P23221	**GADD45A**	0.0055	1.5	0.0016	1.7
A_32_P234459	**HLA-H**	0.0206	1.5	0.0103	1.7
A_23_P350295		0.0009	1.5	0.0003	1.7
A_24_P50489		0.0024	1.5	0.0008	1.5
A_23_P125109		0.0036	1.5	0.0002	1.7
A_23_P313482	**ABRA**	0.0003	1.5	0.0002	1.5

All probes are differentially expressed with a PFP (percentage of false positive) <0.05 and a FC (fold change); −1.5 ≥ FC ≥ 1.5.

**Table 5:** **Down-regulated DEGs between COPD**_L_ **vs. COPD**_N_ **and COPD**_L_ **vs. C with a FC over 1.5**

**PROBE**	**Gene symbol**	**COPD**_**L**_ **vs. COPD**_**N**_		**COPD**_**L**_ **vs. C**	
		**PFP**	**FC**	**PFP**	**FC**
A_24_P401294	**FLJ35934**	0	−1.5	0	−1.5
A_24_P96961	**SPSB1**	0.0076	−1.5	0.0004	−2.2
A_24_P576591		0.0206	−1.5	0.0096	−1.5
A_24_P319675	**RAB10**	0.0105	−1.5	0	−2.8
A_23_P57089	**PMEPA1**	0.0056	−1.5	0.0005	−1.8
A_23_P146339	**GPT**	0.0013	−1.6	0.0007	−1.6
A_23_P308763	**FARP1**	0.0047	−1.6	0.0018	−1.7
A_24_P419028	**MOP-1**	0.0124	−1.6	0.0013	−1.8
A_23_P37856	**HBA2**	0.0003	−1.6	0	−2.4
A_23_P205355	**SERPINA5**	0.0377	−1.7	0.0031	−2.2
A_24_P413126	**PMEPA1**	0.0064	−1.7	0.0006	−1.9
A_24_P368943	**EVX1**	0.0006	−1.7	0.0002	−1.7
A_23_P26457	**HBA2**	0.0005	−1.8	0	−2.5
A_32_P163891		0.0344	−1.8	0.0104	−2.4
A_24_P75190	**HBD**	0.0003	−1.9	0	−3.1
A_24_P20795	**IRX4**	0.0055	−1.9	0.005	−1.9
A_23_P13548	**CHRDL2**	0.0139	−2.0	0.0452	−1.6
A_24_P412734	**PRSS36**	0.0474	−2.3	0.0039	−1.9
A_24_P237328	**MCAT**	0.0483	−2.4	0.0123	−1.8
A_24_P335092	**SAA1**	0.0207	−2.6	0.0003	3.8

All probes are differentially expressed with a PFP (percentage of false positive) < 0.05 and a FC (fold change); −1.5 ≥ FC ≥ 1.5.

### Relationship with muscle structure and function

In order to identify *bona fide* muscle wasting associated genes we selected, from this subset of 42 DEGs, those that correlated with FFMI in the whole population including the healthy control. Ten out of twenty six of the up-regulated DEGs were negatively correlated with FFMI while nine out of the sixteen down-regulated DEGs were positively correlated with FFMI (Table 6).

**Table 6:** **DEGs between COPD**_L_ **and both COPD**_N_ **and C that that varied with percentage of FFMI**

**Source**	**Probe**	**Gene symbol**	**rho**	**p**
**Up-regulated genes**	A_23_P22735	BEX2	−0.55	0.0019
	A_23_P100711	PMP22	−0.48	0.0085
	A_23_P166109	FLRT3	−0.4	0.0327
	A_24_P193295	RAB15	−0.44	0.0167
	A_23_P46426	CYR61	−0.38	0.0391
	A_24_P370946	CYR61	−0.39	0.0358
	A_23_P46429	CYR61	−0.39	0.0337
	A_24_P261734	SLC38A1	−0.47	0.0098
	A_23_P363399	SLC38A1	−0.47	0.01
	A_23_P19733	SLC22A3	−0.55	0.002
	A_23_P49338	TNFRSF12A	−0.36	0.05
	A_32_P219135		−0.45	0.015
	A_23_P34915	ATF3	−0.36	0.055
	A_23_P161218	ANKRD1	−0.47	0.0099
**Down-regulated genes**	A_23_P13548	CHRDL2	0.44	0.017
	A_24_P20795	IRX4	0.36	0.056
	A_24_P368943	EVX1	0.36	0.057
	A_24_P413126	PMEPA1	0.52	0.0034
	A_23_P57089	PMEPA1	0.57	0.0012
	A_23_P205355	SERPINA5	0.5	0.0054
	A_24_P419028	MOP-1	0.37	0.045
	A_23_P146339	GPT	0.36	0.054
	A_24_P319675	RAB10	0.64	0.0002
	A_24_P401294	FLJ35934	0.44	0.0157

Significant associations between up-regulated and down-regulated DEGs and FFMI. rho: spearman correlation index.

Since COPD_L_ were characterized by type II fibre atrophy in comparison with COPD_N_, we explored the correlations between these DEGs and type II fibre area. Three of the up-regulated DEGs (PMP22, CGREF1 and LPL) were negatively correlated with type II fibre area while four of the down-regulated DEGs (SAA1, CHRLD2, and FARP1) were positively correlated with type II fibre area (Additional file 3: Table S1). In turn, several of the up-regulated (ANKRD1, CDKN1A, GADD45A, ATF3, RAB15, ABRA and SLC22A3) and down-regulated (EVX1, PMEPA1, GPT, RAB10, SPSB1) DEGs in COPD_L_ correlate with the proportion of Type II fibres (Additional file 4: Table S2). Moreover several up-regulated (negatively) and down-regulated (positively) DEGs correlated with QMVC, a measurement of muscle function (Additional file 5: Table S3).

**Figure 3:**
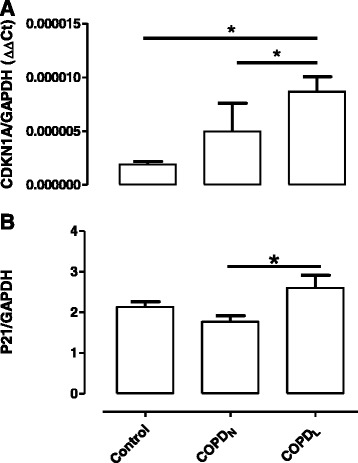
**qPCR (panel A) and Immunoblotting (panel B) results for CDKN1A and p21 respectively showing differential expression in COPD**_L_ **)** (*p < 0.05).

### qPCR validation

Eleven genes (CDKN1A, CEBPA, CYR61, EFCAB7, EGR1, HMOX1, PDE11A, SAA1, SLC22A3, SLC38A1 and SLC43A2) were selected for qPCR validation of the microarray experiment based on either a microarray-derived fold-change (>2) or statistical significance for differential expression and/or the biological relevance for the different comparisons (COPD_N_ vs COPD_L_, COPD_N_ vs C and COPD_L_ vs C) (Figure 3A and Additional file 6: Figure S2). As shown in Table 7, the altered expression of all these genes was confirmed by real time TaqMan PCR.

**Figure 4:**
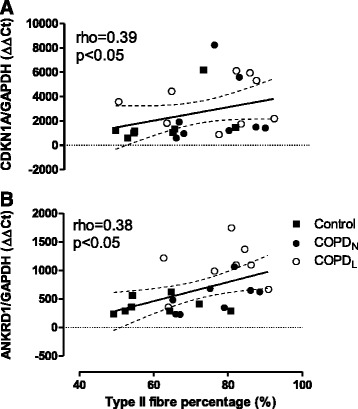
**Correlations between percentage of Type II fibres (x axis) and qPCR gene expression (corrected by GAPDH housekeeping gene (ΔΔCt) CDKN1A panel A; ANKRD1 panel B) in COPD**_L_ **(o), COPD**_N_ **(●) and C (■) (y axis).** Solid line represents regression line and dashed lines 95% CI.

**Table 7:** **qPCR Validation of microarray experiment**

**Gene symbol**	**Microarray selection criteria**	**qPCR result**
CDKN1A	PFP <0.005, FC 2.19 COPD_L_ vs C	p < 0.005
CEBPA	PFP <0.005, FC 2.08 COPD_L_ vs C	p < 0.005
CYR61	PFP <0.005, FC 2.6 COPD_L_ vs C	p < 0.005
EFCAB7	PFP <0.005, FC 2.63 COPD_L_ vs C	p < 0.005
EGR1	PFP <0.005, FC −2.11 COPD_N_ vs C	p < 0.05
HMOX1	PFP <0.005, FC −2.31 COPD_L_ vs C	p = 0.06
	PFP <0.005, FC −2.26 COPD_N_ vs C	p < 0.05
PDE11	PFP <0.005, FC −2.28 COPD_L_ vs C	p < 0.05
SAA1	PFP <0.005, FC 3.8 COPD_L_ vs C	p < 0.005
	PFP <0.005, FC 10.1 COPD_N_ vs C	ns
SLC22A3	PFP <0.005, FC 2.46 COPD_L_ vs COPD_N_	ns
	PFP <0.005, FC 4.62 COPD_L_ vs C	p < 0.05
SLC38A1	PFP <0.005, FC 3.02 COPD_L_ vs C	p < 0.005
SLC43A2	PFP <0.005, FC −2.1 COPD_L_ vs C	p < 0.005

Comparative results of eleven selected genes for validation between microarrays and qPCR.

**Figure 5:**
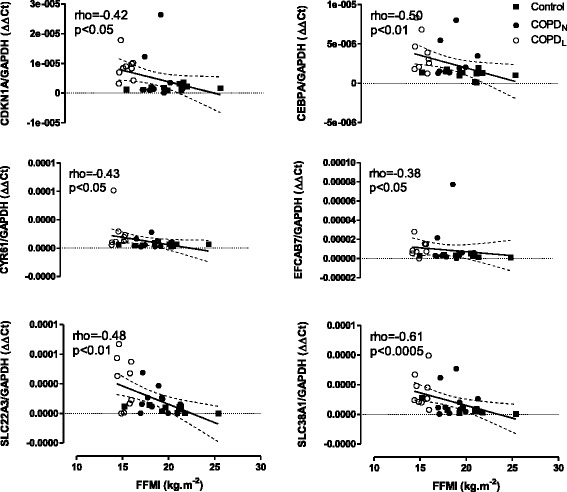
**Correlations between FFMI (x axis) and qPCR gene expression corrected by GAPDH housekeeping gene (ΔΔCt) in COPD**_L_ **(o), COPD**_N_ **(●) and C (■) (y axis).** Solid line represents regression line and dashed lines 95% CI.

Figure 3A shows qPCR data for CDKN1A, one of the six genes representing the group of genes related to cell cycle inhibition and inhibition of cell growth from the list of 42 DEGs identified between COPD_L_ and both other groups of subjects with normal muscle mass. CDKN1A mRNA expression was increased in COPD_L_ compared to both COPD_N_ and C (p < 0.005) (Graphics for the remaining eight genes are depicted in the Additional file 6: Figure S2). The expression of several of these genes assessed by qPCR correlated with parameters of muscle structure and function. CDKN1A and ANKRD1 correlated with percentage of Type II fibres (Figure 4), CEBPA, CYR61, EFCAB7, SLC22A3 and SLC38A1 correlated negatively with FFMI (Figure 5). In turn, CEBPA correlated positively (rho = 0.45, p < 0.05) while SLC43A2 correlated negatively (rho = −0.65, p < 0.0005) with the proportion of Type II fibres. Moreover, CDKN1A, CYR61, CEBPA, EFCAB7, SLC22A3 correlated negatively with QMVC, a measurement of muscle function (Additional file 7: Figure S3).

### Immunobloting

As CDKN1A was differentially expressed between COPD_L_ and COPD_N_ but also between COPD_L_ and C, with a fold change over 1.5, and it also correlated with several measurements of skeletal muscle structure and function and is consistently overexpressed in other conditions characterised by muscle wasting, we chose to explore the protein levels of p21 in our samples by immunoblotting. This showed that p21 is over expressed in COPD_L_ in comparison to both COPD_N_ and C (Figure 3B). However, only the comparison between COPD_L_ and COPD_N_ was statistically significant.

## Discussion

This study shows relevant changes in gene expression in the *vastus lateralis* of COPD patients with skeletal muscle wasting. We identified several up-regulated genes associated with cell cycle arrest and growth regulation, and down-regulated genes associated with muscle formation and glucose metabolism in patients with low FFMI. These DEGs correlated with several parameters of muscle structure and function. The altered expression of 11 genes was confirmed by qPCR and the increased level in COPD_L_ of P21, the protein encoded by CDKN1A, was also corroborated by immunoblotting.

Our initial approach revealed extensive changes in gene expression that allowed us to identify several biologically relevant GO terms related to protein synthesis, muscle contraction and muscle organ development (from the up-regulated list of genes in COPD_L_) and oxidative energy production, glucose metabolism and striated muscle contraction and myogenesis (from the down-regulated list of genes in COPD_L_). The concomitant activation and deregulation of different genes involved in myogenesis reveal the complex nature of the muscle wasting process in this population of patients. This, together with the activation of genes related to protein synthesis, show a potentially adaptive, yet clearly ineffective, response at maintaining muscle mass [37].

Using a more restrictive approach we were able to discard genes with potential marginal changes and identify a subset of more substantial DEGs. This group of DEGs fulfil several criteria: a) they were differentially expressed between COPD_L_ and COPD_N_, b) showed a fold change ≥1.5 or ≤1.5 between COPD_L_ and COPD_N_, c) were also differentially expressed between COPD_L_ and C, b) showed a fold change ≥1.5 or ≤1.5 between COPD_L_ and C. These criteria markedly reduced the list of DEGs but reinforced the relevance of these genes for the process of muscle wasting. This approach identified up-regulated DEGs related to the inhibition of cell growth and cell cycle and down-regulated genes related to myogenesis and glucose metabolism in COPD_L_. Several of these DEGs correlate with FFMI, parameters of muscle structure and function such as type II fibre size (the fibres showing atrophy in COPD_L_), the proportion of type II fibres and QMVC, indicating a potential role of these genes in the muscle wasting process.

Among these DEGs, GADD45A is induced by DNA damage [38] and stress (such as fasting or immobilization) [39,40]. GADD45A represses genes involved in anabolic signalling and energy production and induces pro-atrophy genes [39,40]. Interestingly, genes related to energy production by oxidation and glucose metabolic processes were down-regulated in our population of COPD_L_. GADD45A [41] and CDKN1A [42-44] have previously been identified in several conditions associated with muscle atrophy. Moreover, myostatin suppresses muscle cell growth via the transcriptional regulation of CDKN1A [45]. p21, the protein encoded by this gene, can inhibit apoptosis (when present in the cytosol by arresting the cell cycle allowing for DNA repair) or promote apoptosis (in the nucleus and interacting with other proteins) [38,46,47]. The interaction between p21 and growth arrest and DNA damage-inducible genes such as GADD45A results in apoptosis [38]. Furthermore, CDKN1A and GADD45A are also related to other muscular disorders such as amyotrophic lateral sclerosis [43]. ATF3, another of the DEGs up-regulated in COPD_L_, together with GADD45A and CDKN1A, is also associated with cell cycle arrest in response to DNA damage [48]. ATF3 is induced by stress and is related to cardiac contractility abnormalities [49] and muscular disorders such as amyotrophic lateral sclerosis [43]. ATF3 gene expression correlated negatively with FFMI and QMVC in our population. Another gene up-regulated in COPD_L_, ANKRD1, has been associated with up-regulation of p21, one of its downstream targets [50]. ANKRD1 is expressed in the muscle and migrates to the nucleus when the muscle is under stress. It has been shown to be up-regulated during muscle atrophy [51]. Evidence suggests that ANKRD1 per se cannot initiate atrophy. However, the observation that p21 is increased whenever ANDRD1 is increased in different models of muscle atrophy highlights the importance of this interaction in the muscle wasting process [50]. Moreover, a switch towards fast-twitch fibres has been reported in models of atrophy in association with up-regulation of ANKRD1 [50]. It is of note that ANKRD1 expression was negatively correlated with FFMI and QMVC and positively correlated with the proportion of Type II fibres in our COPD_L_, as were CDKN1A, GADD45A1 and ATF3. GADD45A1 also correlated negatively with muscle function measured as QMVC. CDKN1A has also been shown to interact with CEBPA [46] leading to cell arrest [38], and was up-regulated in COPD_L_ and confirmed by qPCR. In fact, CEBPA inhibits cell proliferation by stabilising p21 [52] and protecting against its degradation [53]. CEBPA and CDKN1A gene expression assessed by qPCR correlated negatively with FFMI and QMVC in our population. The simultaneous up-regulation of these genes in COPD_L_ together with the strong association with FFMI, and parameters of skeletal muscle structure and function suggest that cell cycle arrest followed by apoptosis play a role in the process of muscle wasting in this population in response to stress. The presence of muscle cell apoptosis in peripheral muscle of patients with COPD is still controversial. Some groups have reported increased cell apoptosis, assessed as DNA fragmentation [54,55], whereas others failed to report apoptosis, assessed as active caspase-3 [56]. It is interesting that, as mentioned previously, p21 has a dual function and can promote or inhibit apoptosis [38,46,47]. Apoptosis induced by p21 is distinct from that induced by other pro-apoptotic agents and does not involve activation of caspases [38]. In fact, while the carboxyl terminus interacts with molecules such as GADD45A or CEBPA, the N-terminus can interact with procaspase-3 to block activation of caspase-3 [57]. Hence, apoptosis induced by p21 is not affected by caspase inhibitors [38]. This may explain the controversy in the literature regarding muscle cell apoptosis in peripheral muscle of patients with COPD.

Other genes known to be overexpressed under stress situations and inflammation were also up-regulated in COPD_L_, such as CYR61 whose expression increases with exercise in humans [58-60]. CYR61 is required for the migration of myoblasts during the regeneration process [61] and mediates angiogenesis [60,62]. On the other hand, CY61 is increased in the muscle in models of malnutrition [63] and muscle denervation [64] and promotes cell migration and immobilise inflammatory cells in the site of inflammation and tissue repair [65]. Moreover, CYR61 gene expression assessed by both, microarray and qPCR, correlated negatively with FFM and QMVC in our population. In line with these findings, oxidative stress has been consistently shown in limb muscle of patients with COPD [66], particularly in patients with muscle wasting [67]. While local muscle oxidative stress is induced by exercise [67], the exercise-induced increase in antioxidant enzymes is attenuated or inhibited in the muscle of underweight patients with COPD [68,69]. HMOX1, a gene involved in the response to oxidants known to protect against cytotoxicity of oxidative stress and nitric oxide metabolism, was down-regulated in our population of COPD [70]. In turn, although not consistently shown, some authors have demonstrated an increase in local inflammation in the muscle of patients with COPD [71-73]. Regardless of the presence of inflammatory markers, activation of nuclear factor kB (NFkB) in peripheral muscle of patients with COPD suggests an increased inflammatory state [66,74-76]. TNFRS12A, a direct kB target, also known as TWEAK, is known to induce muscle wasting in whole muscle [77] and required for denervation atrophy [78], an effect mediated by NFkB [51,77]. TNFRS12A was up-regulated in COPD_L_ and negatively correlated with FFMI and QMVC in our population.

The maintenance of skeletal muscle bulk results from the interaction of mechanisms leading muscle wasting (i.e. cell death, protein degradation) and muscle regeneration and protein synthesis. Several genes related to muscle development and regeneration (PMEPA1, IRX4, CHRDL2) were down-regulated in the COPD_L_ [79,80]. Some groups have investigated key markers of muscle regeneration in peripheral muscle of patients with COPD, although the evidence is unclear due to the lack of longitudinal data. Plant et al. (80) showed no differences in skeletal muscle expression of muscle-specific transcription factors associated with muscle differentiation Myf5, MyoD or myogenin. Crul et al. [81] showed no differences in MyoD in stable COPD patients. However, patients undergoing an exacerbation presented with reduced levels of MyoD compared to healthy controls (78). Thériault et al. have recently shown that besides greater number of attempts to regenerate the muscle, there was a profound reduction in the differentiation potential in COPD patients peripheral muscle [82]. Vogiatzis et al. (123) showed that exercise training increased the expression of MyoD in peripheral muscle of patents with COPD. Lewis et al. (124) showed an increment in IGF-I protein (an activator of cell growth and proliferation and an inhibitor of apoptosis) with exercise training and a combination of exercise training and testosterone together with an increment in myogenin mRNA expression. The role of impaired muscle regeneration, potentially in some circumstances (e.g. exacerbations), remains to be elucidated.

### Comparison with other studies

We have identified other studies assessing peripheral muscle gene expression in COPD and other medical conditions associated with muscle cachexia, namely intensive care (ICU) patients [83] and cancer [84], as well as ageing-associated muscle wasting [85,86] and studies exploring the effect of exercise training on gene expression in muscle of healthy subjects [87].

Despite differences in the populations and the methodology, which oblige to be cautious when comparing studies, we found interesting similarities with our results.

Two studies have explored gene expression in peripheral muscle of patients with COPD [88,89]. Debigaré [88] et al. compared *vastus lateralis* gene expression profiles from COPD patients with muscle atrophy and healthy controls. They have also found down regulation of genes involved in energy production and up-regulated genes related to cell cycle arrest including CDKN1A in the population of patients with COPD and muscle wasting. In contrast, while several relevant genes from our study were also identified by other studies involving stable COPD patients [88] this was not the case in studies involving hospitalized patients [89], however, as in our study, pro-inflammatory genes, such as SAA, were identified in the exacerbated patients.

In addition, our results have similarities with other inflammatory medical conditions associated with muscle wasting, such as sepsis [83], and also in association with ageing [85,86]. Similarly to our population, genes encoding proteins involved in muscle metabolism are down-regulated in association with ageing. Moreover, genes involved in cell cycle regulation and apoptosis are also affected by age. CDKN1A was up-regulated in the two mentioned studies in association with ageing. The similarities between our findings and studies exploring gene expression in ageing suggest that cell senescence may play a role as a pathogenic mechanism of muscle wasting in COPD. In fact, animal models of premature ageing show structural changes in the lungs and skeletal muscle that resemble those in COPD [90]. Shortening of telomeres, a marker of premature ageing, has been described in patients with COPD and muscle wasting [21].

Interestingly, as shown by Fredriksson et al. [83] in septic patients admitted to an intensive care unit (ICU), pro-inflammatory genes are up-regulated in the muscle of these patients including several genes found in our study. Moreover, they also found up-regulated genes related to cell cycle arrest including CDKN1A suggesting that that cell senescence could be also a mechanism leading to muscle wasting in this population. Moreover, inflammation and oxidative stress, a feature of patients with COPD and muscle wasting has been associated with stress-induced premature senescence [91].

Interestingly, when exploring the effects of exercise in skeletal muscle gene expression [86,87], while exercise training contribute to “normalise” the expression of genes related with energy production and oxidative capacity of the muscle, several genes found up-regulated in our COPD population were not modified (“normalized”) by exercise training (including GADD45A, NNMT, ANKRD1, ATF3 and SLC38A1) suggesting that the increased expression of these genes in our population of COPD_L_ is not related to physical activity levels since this should produce the opposite effect.

It is of note that the gene profile of patients with cancer [84] was not comparable to the profile of patients with COPD and muscle wasting of our study proposing the possibility that muscle wasting in these two conditions involve different underlying mechanisms.

### Limitations of the study

We have used FFMI measured by bioimpedance as a surrogate of muscle mass instead of a direct measurement of muscle mass. One of the most validated definitions of sarcopenia and severity of sarcopenia is based in BIA assessment of muscle mass [92]. This method has been validated against body composition assessed by deuterium dilution [93] and dual-energy X-ray absorptiometry [94] and shows very good correlation with other measurements of limb muscle mass in patients with COPD [95]. FFMI measured by bioimpedance is an independent predictor of skeletal muscle function and exercise capacity [96] and mortality [97] in patients with COPD. Moreover, it is a very sensitive method to detect undernutrition in these patients [98]. Values to differentiate muscle wasted patients from patients with preserved muscle mass based of FFMI have been established and validated in patients with COPD [93]. In our population, FFMI related to muscle function assessed as QMVC.

Physical activity (PA) can be seen as a confounding variable in this study. It was assessed with two questionnaires instead of using a direct measurement (i.e. activity monitors). PA assessed with the Voorrips questionnaire, designed to be use in elderly populations, showed lower physical activity levels in COPD_L_. It was difficult to recruit patients with higher levels of PA assessed with this tool. Interestingly, there was no difference in the level of Activity of Daily Living assessed with the London Chest Activity of Daily Living Scale. Moreover, key DEGs identified in the present study showed no modification in response to exercise training in young [87] and elderly [86] subjects making it less likely that these changes were attributed to a lower PA level in this population.

Beside the efforts in matching the populations, healthy control subjects present differences in average cessation days and Pack/years in comparison to COPD_L_. Environmental factors such as cigarette smoking may associate with peripheral muscle alterations [99]. However, it is unlikely to explain the differences in the transcriptome in our population as all three groups were matched for smoking status. Moreover, only one COPD_L_ was an active smoker, whilst the other two groups included two active smokers each, which makes it less possible that this is a conditioning factor for muscle wasting in this group. Furthermore, no differences in the average cessation years, in cumulative history of smoking nor in age at smoking cessation (which may have important implications on outcomes) [100] were seen between COPD_L_ and COPD_N_. Therefore, it is unlikely that the time post-smoking cessation contributes to the differences in gene expression between these groups.

## Conclusions

This study demonstrate that *vastus lateralis* of patients with COPD and muscle wasting overexpress genes related to inhibition of cell cycle and of cell growth while genes related to muscle formation and growth and energy production were down-regulated. This pattern is similar to observations associated with ageing which, suggests that premature ageing may play a role in muscle atrophy in COPD. This profile, together with several genes involved in inflammation signaling, were shared with the profile described in severely ill patients in the ICU which suggest both, that ICU patients may also experience cell senescence in response to inflammation and that inflammation may be a shared mechanism between COPD and ICU patients. These results may open new avenues for the treatment of muscle wasting in patients with COPD. The most challenging issue is to explore potential avenues for treatment and identify the timing to treat these with anti-aging agents considering that earlier diagnosis is a key for effective anti-aging therapy.

## Additional files

Additional file 1:
**Methods.**
Additional file 2: Figure S1. Lung Function and Smoking History. Lung Function and Smoking History in COPDL, COPDN and C. (*p < 0.05). Additional file 3: Table S1. DEG between COPDL and both COPDN and C which varied with fibre type II area. List of up and down DEG genes between COPDL and both COPDN and C that varied with type II area in the whole populations. Additional file 4: Table S2. DEG between COPDL and both COPDN and C which varied with fibre type II percentage. List of up and down DEG genes between COPDL and both COPDN and C that varied with type II fibre percentage in the whole populations. Additional file 5: Table S3. DEG between COPDL and both COPDN and C which varied with fibre muscle function measured as QMVC. List of up and down DEG genes between COPDL and both COPDN and C that varied with QMVC in the whole populations. Additional file 6: Figure S2. qPCR graphics for the validated genes between COPDL and both other groups COPDN and C. qPCR validated genes in COPDL, COPDN and C. (*p < 0.05). Additional file 7: Figure S3. Correlations between qPCR genes and QMVC. Correlations between QMVC (x axis) and qPCR gene expression corrected by GAPDH housekeeping gene (ΔΔCt) in COPDL (o), COPDN (●) and C (■) (y axis). Solid line represents regression line and dashed lines 95% CI.

## Abbreviations

mRNA: m Ribonucleic acid
COPD: Chronic obstructive pulmonary disease
FFMI: Fat free mass index
COPDL: Patients with COPD and low FFMI
COPDN: Patients with COPD and normal FFMI
C: Control subjects
HRQoL: Health related quality of life
BIA: Bioimpedance analysis
6MWD: Six minute walking distance
QMVC: Quadriceps maximal voluntary contraction
SGRQ: Saint George’s respiratory questionnaire
mMRC: Modified medical research council dyspnoea score
PA: Physical activity
PA_V_: Physical activity assessed with the Voorrips questionnaire
LCADL: London chest activity of daily living scale
PA_L_: Physical activity assessed with the LCADL
PFP: Percent false positives
FDR: False discovery rate
GO: Gene ontology
qPCR: Real time polymerase chain reaction
ΔCT: Delta cycle threshold
ADL: Activities of daily living
FEV_1_: Forced expiratory volume in the first second
FVC: Forced vital capacity
RP: Rank products
DEGs: Differentially expressed genes
FC: Fold change
DNA: Deoxyribonucleic acid
ICU: Intensive care unit
PaO_2_: Arterial oxygen partial pressure
PaCO_2_: Arterial carbon dioxide partial pressure
