# Supplementary material for: Genome-wide mRNA expression profiling in vastus lateralis of COPD patients with low and normal fat free mass index and healthy controls
Source: Respir Res. 2015 Jan 8;16(1):1. doi: 10.1186/s12931-014-0139-5 (PMC4333166; doi:10.1186/s12931-014-0139-5)
Supplement: Additional file 1: — Methods. [file 12931_2014_139_MOESM1_ESM.pdf]

METHODS

Study Group

Ten clinically stable COPD patients (8 male) with normal FFMI ($COPD_N$), ten COPD patients (8 male) with low FFMI ($COPD_L$) and ten age gender and smoking status-matched healthy sedentary subjects (8 male) were included in the present study (**Table 1**). COPD patients had a history compatible with the disease, at least 10 packyears of smoking and evidence of chronic airflow limitation (FEV_1 / FVC < 0.7, FEV_1 <80% predicted). Subjects were considered ex-smokers if they had not smoked for at least 6 month otherwise were considered active smokers. No never-smoker were included in the study.

All patients were treated with short- and long-acting bronchodilators and inhaled corticosteroids. No patients were taking oral corticosteroids or drugs with potential effect on the muscle. They were clinically stable at the time of the study, without an exacerbation or oral steroid treatment in the previous six weeks. None of the patients had significant co-morbidities.

Measurements to characterize the subjects included: a) Clinical assessment, b) Smoking status and cumulative smoking history, c) Spirometry and blood gases, d) Body composition by bioimpedance analysis (BIA), e) Maximal isometric quadriceps maximal voluntary contraction (QMVC), e) exercise tolerance-6 minute walking distance, f) Health related quality of life (HRQoL) (St George's Respiratory Questionnaire), g) number of exacerbations in the previous year, h) Activities of daily living (London Chest Activity of Daily Living Scale [LCADL]) questionnaire; and i) Physical activity questionnaire (Voorrips).

All participants were informed of any risks and discomfort associated with the study, and written informed consent was obtained. The study was approved by the Lothian Regional Ethics Committee. Some of the results of these studies have been previously reported in the form of an abstract [1, 2].

MEASUREMENTS

Lung function

Spirometry was measured (Alpha Spirometer; Vitalograph, Buckingham, UK) according to American Thoracic Society/European Respiratory Society standards in all subjects [3] before and after the administration of 2.5 mg of nebulised salbutamol. Arterial oxygen tension (PaO_2), carbon dioxide tension ($PaCO_2$), bicarbonate (HCO_3^-) and pH were analyzed on a blood gas analyzer (Ciba Corning 800, USA).

Body composition

Body composition was estimated by a leg-to-leg bioelectric impedance device (TBF-300M, TANITA Corporation, Tokyo, Japan) while subjects were in supine position. Fat free mass index was obtained by dividing FFM in Kg by height^2. Low FFMI was defined as <16 kg.m^{-2} for male and < 15 kg.m^{-2} for female COPD patients [4].

Exercise tolerance and muscle strength

As a measure of exercise tolerance all 30 participants in the present study performed an encouraged 6MWT according to ATS guidelines [5]. As a

measure of muscle function [6], muscle strength was assessed as the maximal isometric quadriceps voluntary contraction (QMVC) using a strain gauge dynamometer (Chatillon® K-MSC 500, Ametek, Florida). Subjects were asked to sit in a purpose-built chair with an inextensible strap connecting the ankle to a strain gauge; knees were flexed to 90°; the strain gauge and couplings were all aligned to ensure that the contraction was isometric. After a previous muscle warm up QMVC was performed 3 to 4 times with vigorous encouragement and rests between contractions; the biggest effort recorded was used for analysis.

Health related quality of life (HRQoL) and physical activity level

HRQoL was assessed using the St. Geroge's Respiratory Questionnaire [7]. Physical activity level was assessed using the Voorrips physical activity questionnaire in the whole population participating in the study [8]. Moreover, COPD patients activities of daily living was assessed specifically with the London Chest Activity of Daily Living Scale (LCADL [9].

***Vastus Lateralis* muscle biopsy and RNA isolation**

An open muscle biopsy of the *"vastus lateralis"* was obtained under local anesthetic and ~0.1 g was included in RNA stabilization reagent (RNAlater®, Ambion, Inc., USA) and stored at -20°C for RNA extraction. Total RNA was extracted and purified by homogenisation (TissueLyser, Qiagen Ltd. West Sussex, UK) of tissue and employing the TRIzol® Plus RNA Purification Kit (Invitrogen Life Technologies, Carlsbad, CA) according to the manufacturer's

protocol. The quality of RNA samples was evaluated by capillary electrophoresis using an Agilent 2100 Bioanalyzer (Agilent Technologies, USA). The remaining sample was aliquoted and stored at -80°C.

Fibre type typification

Paraffin sections (5um) were de-waxed and re-hydrated through graded ethanol using standard procedures. Sections were placed in 250 ml of Novocastra pH8 retrieval buffer and subjected to antigen retrieval in a de-cloaking chamber (Biocare Medical, USA) using a protocol described elsewhere [10]. Briefly, sections were heated to 125 ^{o}C for 30 s and allowed to cool to 90 ^{o}C for 10 s. Sections were washed in running tap water then placed on a Leica Vision Biosystems Bond max immmunostaining robot and stained as follows. Sections were incubated with 3% H_2O_2 for 10 min washed in water then TBS/Tween 0.1%. Following incubation with 20% Normal goat serum in TBS/Tween the sections were incubated for 120 min with mouse anti myosin Type I at 1:2000 dilution (Dako), following further washes slides were incubated with goat anti Mouse fab peroxidase at 1:500 in NGS/Tween for 30 min. Following washing slides were incubated with Tyramide Cy5 (Blue) (Perkin Elmer) for 10 min and washed. Using antibody elution using HIER as described by Toth et al [10] sections for Type II co-localisation were retrieved for 10 min in Bond ER_2 epitope retrieval solution followed by 15 min in 0.5mg/ml trypsin (Sigma) in Tris/$CaCl_2$ buffer at 37 C. Sections were incubated with 3% H_2O_2 for 10 min washed in water then TBS/Tween. Following incubation with 20% Normal goat serum in TBS/Tween the sections

were incubated for 120 min with mouse anti myosin Type II (1:5000) following further washes slides were incubated with goat anti Mouse fab peroxidase at 1:500 in NGS/Tween for 30 mins. Following washing, slides were incubated with Tyramide Cy3 (Red) (Perkin Elmer) for 10 min and counterstained with DAPI. Tiled images of the entire section were acquired using a Zeiss 710 confocal microscope. Five images per patient were included in the analysis. A total of 959.6±146.4; 715.0±89.2 and 918.6±95.2 fibres in Control subjects, $COPD_N$ and $COPD_L$ respectively (p=ns) were assessed. Type I, Type II and hybrid (identified by the two antibodies) fibres were counted using a manual tag protocol using Media Cybernetics Image pro Plus (Image-Pro Plus , Media Cybernetics, Inc. Bethesda, MD. USA) and expressed as a proportion of total fibres assessed.

Microarray hybridization and data analysis

Five hundred nanograms of total RNA corresponding to the 30 target samples (10 $COPD_L$, 10 $COPD_N$ and 10 Healthy controls) was converted into labelled cRNA with nucleotides coupled to a fluorescent dye (Cy3) using the Quick Amp Kit (Agilent Technologies, Palo Alto, CA) following the manufacturer's protocol. Samples sizes of 10 provide 80% power for detection of 2-fold changes in expression[11]. Cy3-labeled cRNA (1.65 µg) was hybridized to Agilent Human Whole Genome 4x44K Microarrays (Agilent Technologies, Santa Clara, CA). The hybridized array was then washed and scanned and the data were extracted from the scanned image using Feature Extraction version 10.2 (Agilent Technologies).

Pre-processing (background correction, normalization, filtering and summarization) subsequent data processing and analysis was performed using the Agi4x44 Pre-process module from *bioconductor*[12, 13]. One of the $COPD_L$ patients RNA sample showed very low signal and so was excluded from the analysis.

A non-parametric alternative to the conventional pairwise t-test, the Rank Products[14-16] (RP), was employed. This algorithm is particularly powerful for noisy data and low numbers of replicates. RP detects probes which, when ranked by fold-change between samples from different groups, consistently appear high on the list. The relatively weak assumptions employed by this method make it robust to high levels of noise. RP employs a 'percent false positives' (PFP) measure that can be used to select the most significant differential expressions. A percentage of false positive (PFP) below 0.05 was considered statistically significant.

The gene functional enrichment analysis was performed using DAVID Bioinformatics Resources (http://david.abcc.ncifcrf.gov/)[17]. Specifically, the Functional Annotation Chart tool was used to enrich the over-represented Gene Ontology (GO) terms among the differentially expressed gene list. A list of all detectable transcripts was used as the background for the GO analysis [18]. The GO terms after correction for FDR at $P < 0.05$ (Benjamini Hochberg) were selected for further analysis and interpretation.

Ingenuity Pathway Analysis (IPA, Ingenuity Systems, Redwood, CA) [19].was used to further investigate the Genespring expression clusters. IPA is a proprietary online software tool that aids researchers in modeling and

analyzing the biological significance of microarray datasets. IPA can take data analysis a step further by using the Ingenuity Knowledge Base to identify statistically significant canonical pathways. The Knowledge Base consists of millions of relationships (between genes, proteins, small molecules, and complexes) manually extracted from PhD-level scientists from over 200,000 peer-reviewed articles [19]. Furthermore, close to 3,000 articles in the biological literature have cited this analysis tool (http://www.ingenuity.com/library/pdf/bibliography.pdf). The significance of the association between the genes in each dataset and the canonical pathway was determined by using Fischer's exact test to calculate a *P* value determining the probability that the association between the genes in the dataset and the canonical pathway was explained by chance alone.

All data collected and analysed in this study adhere to the MIAME guidelines and all primary microarray data will be submitted to one of the public repositories in a format that complies with the MIAME guidelines.

qPCR validation

Given the size of the study and inherent variation in patient samples for microarray studies, relevant genes identified in the analysis were also validated by quantitative reverse transcriptase-polymerase chain reaction (pPCR). Based on microarray-derived fold-change (>2) or statistical significance for differential expression and/or the biological relevance for the different comparisons ($COPD_N$ vs $COPD_L$, $COPD_N$ vs C and $COPD_L$ vs C), eleven genes (CDKN1A, CEBPA, CYR61, EFCAB7, EGR1, HMOX1,

PDE11A, SAA1, SLC22A3, SLC38A1 and SLC43A2) were selected for qPCR validation of the microarray experiment. Pre-develop assays were selected using the UMapIt Microarray-to-TaqMan assays mapping tool (Applied Biosystems). This tool enables a search for the most appropriate pre-desiged TaqMan assay relative to the probe from the microarray. cDNA was synthesized from 1 ug of total RNA (from the same samples used for microarray experiments) using the High-Capacity cDNA Archive kit and PCR reactions were assembled with TaqMan Universal PCR Master Mix following the manufacturer's protocol (Applied Biosystems) in an array format. Samples were analyzed in duplicate on a *7900HT Fast Real-Time PCR System* (Applied Biosystems) in an array format, and Ct values were obtained from the *ABI PRISM® 7900 Sequence Detection System* (SDS version 2.1) software. Ct values were subsequently normalized using the 18S ribosomal subunit gene as an internal, endogenous control. These ΔCt values [Ct (gene) – Ct (18S)] were generated with the RealTime StatMiner® Software (Integromics) which was also used for the quality control of the arrays. One sample was detected as outlier and was excluded from the analysis. This sample corresponds to the same outlier detected in the microarray experiment.

Immunoblotting

CDKN1A was determined using immunoblotting. 20 μg protein, as determined by the BCA protein micro assay (BioRAD, Hercules, CA), were resolved by sodium-dodecyl sulfate-polyacrylamide gel electrophoresis on 10%

polyacrylamide gels. Proteins were transferred to Immobilon-P PVDF membranes (Millipore, Billerica, MA), blocked with 5 % dry milk (Bio-Rad, München, Germany) in TBS (Sigma) overnight at $4^{o}C$ and probed with primary antibodies against CDKN1A (ab 7960) (Abcam, Bristol, UK) during 1 h at room temperature. Proteins were then visualized using the ECL Detection System (Pierce, Rockford, IL) as per the manufacturer's instructions.

Statistical analysis

Anthropometric, physiological data and immunoblotting results for CDKN1A are expressed as mean±SEM. These data were analysed using ANOVA with Student-Newman-Keuls as a post-hoc test.

Correlation analysis between variables was conducted using Pearson's correlation index for continuous variables and Spearman's correlation index for categorical variables. For the qPCR validation analysis differential expression analysis on individual sample values of ΔCT using Kruskal Wallis with a Nemenyi-Damico-Wolfe-Dunn post-hoc test was performed.

The data were analyzed using the statistical package program SAS version 9.3 (SAS Institute Inc, Cary, NC, USA). A p value <0.05 was taken as statistically significant.

RESULTS

TABLE S1. DEG between $COPD_L$ and both $COPD_N$ and C that that varied with fibre type II area.

Source	Probe	Gene Symbol	rho	p
Up-regulated genes	A_23_P100711	PMP22	-0.44	0.027
	A_23_P403445	CGREF1	-0.60	0.0013
	A_23_P146233	LPL	-0.42	0.032
Down-regulated genes	A_24_P335092	SAA1	0.49	0.012
	A_23_P13548	CHRLD	0.39	0.05
	A_23_P308763	FARP1	0.48	0.015
	A_24_P401294	FLJ35934	0.44	0.029

Table S1. *List of up and down DEG genes between $COPD_L$ and both $COPD_N$ and C that varied with type II area in the whole populations.*

TABLE S2. DEG between $COPD_L$ and both $COPD_N$ and C that that varied with fibre type II percentage.

Source	Probe	Gene Symbol	rho	p
Up-regulated genes	A_23_P59210	CDKN1A	0.39	<0.05
	A_23_P23221	GADD45A	0.47	<0.05
	A_23_P19733	SLS22A3	0.59	<0.005
	A_23_P34915	ATF3	0.43	<0.05
	A_23_P313482	ABRA	0.41	<0.05
	A_23_P161218	ANKRD1	0.38	<0.05
	A_24_P193295	RAB15	0.45	<0.05
Down-regulated genes	A_23_P57089	PMEPA1	-0.46	<0.05
	A_24_P319675	RAB10	-0.40	<0.05
	A_23_P146339	GPT	-0.46	<0.05
	A_24_P96961	SPSB1	-0.54	<0.01
	A_24_P368943	EVX1	-0.39	<0.05

Table S2. *List of up and down DEG genes between $COPD_L$ and both $COPD_N$ and C that varied with type II fibre percentage in the whole populations.*

TABLE S3. DEG between $COPD_L$ and both $COPD_N$ and C which varied with fibre muscle function measured as QMVC.

Source	Probe	Gene	rho	p
Up-regulated genes	A_32_P234459	HLA-H	-0.46	<0.05
	A_23_P23221	GADD45A	-0.44	<0.05
	A_23_P22735	BEX2	-0.41	<0.05
	A_24_P193295	RAB15	-0.61	<0.005
	A_23_P403445	CGREF1	-0.39	<0.05
	A_23_P46426	CYR61	-0.58	<0.05
	A_24_P370946	CYR61	-0.60	<0.005
	A_23_P46429	CYR61	-0.59	<0.005
	A_24_P261734	SLC38A1	-0.44	<0.05
	A_23_P363399	SLC38A1	-0.45	<0.05
	A_23_P19733	SLC22A3	-0.41	<0.05
	A_23_P49338	TNFRSF12A	-0.50	<0.01
	A_23_P127584	NNMT	-0.39	<0.05
	A_32_P60459	OTUD1	-0.44	<0.05
	A_23_P34915	ATF3	-0.40	<0.05
	A_23_P161218	ANKRD1	-0.63	<0.0005
	A_32_P200144	IGHG1	-0.39	<0.05
Down-regulated genes	A_24_P413126	PMEPA1	0.48	<0.05
	A_23_P57089	PMEPA1	0.58	<0.005
	A_23_P146339	GPT	0.57	<0.005
	A_24_P96961	SPSB1	0.50	<0.01

Table S3. *List of up and down DEG genes between $COPD_L$ and both $COPD_N$ and C that varied with QMVC in the whole populations.*

Figure S1. Lung Function and Smoking History.

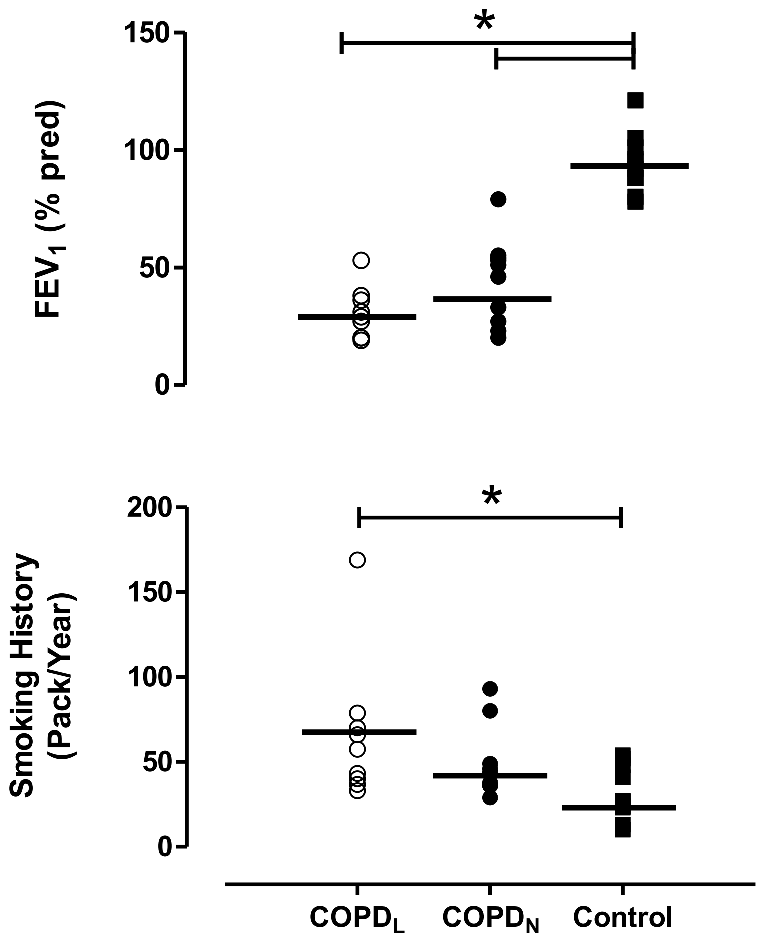

Figure S1: Lung Function and Smoking History in $COPD_L$, $COPD_N$ and C. (*p<0.05).

Figure S2. qPCR graphics for the validated genes between $COPD_L$ and both other groups $COPD_N$ and C.

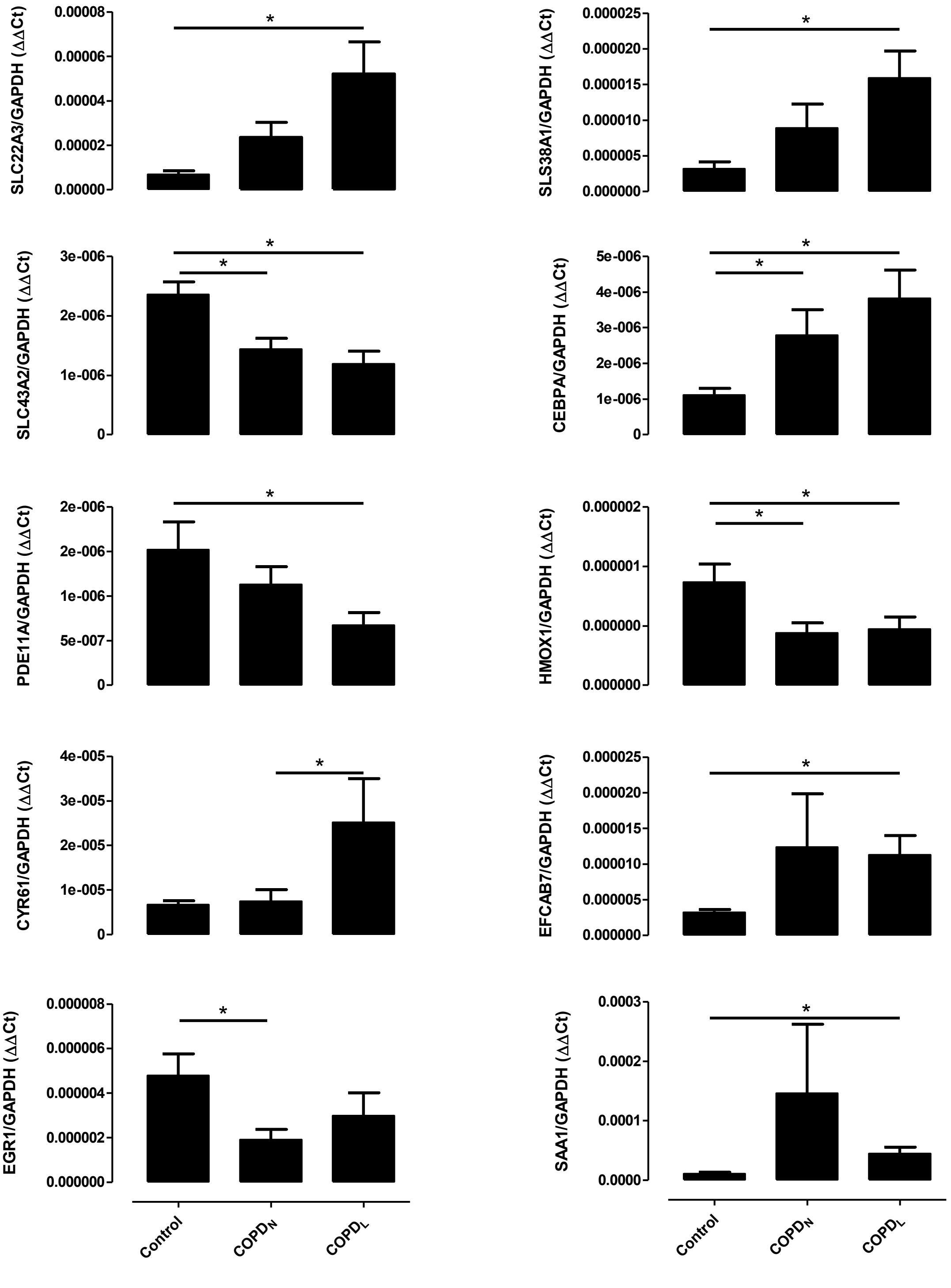

Figure S2: qPCR validated genes in $COPD_L$, $COPD_N$ and C. (*$p<0.05$).

Figure S3. Correlations between qPCR genes and QMVC

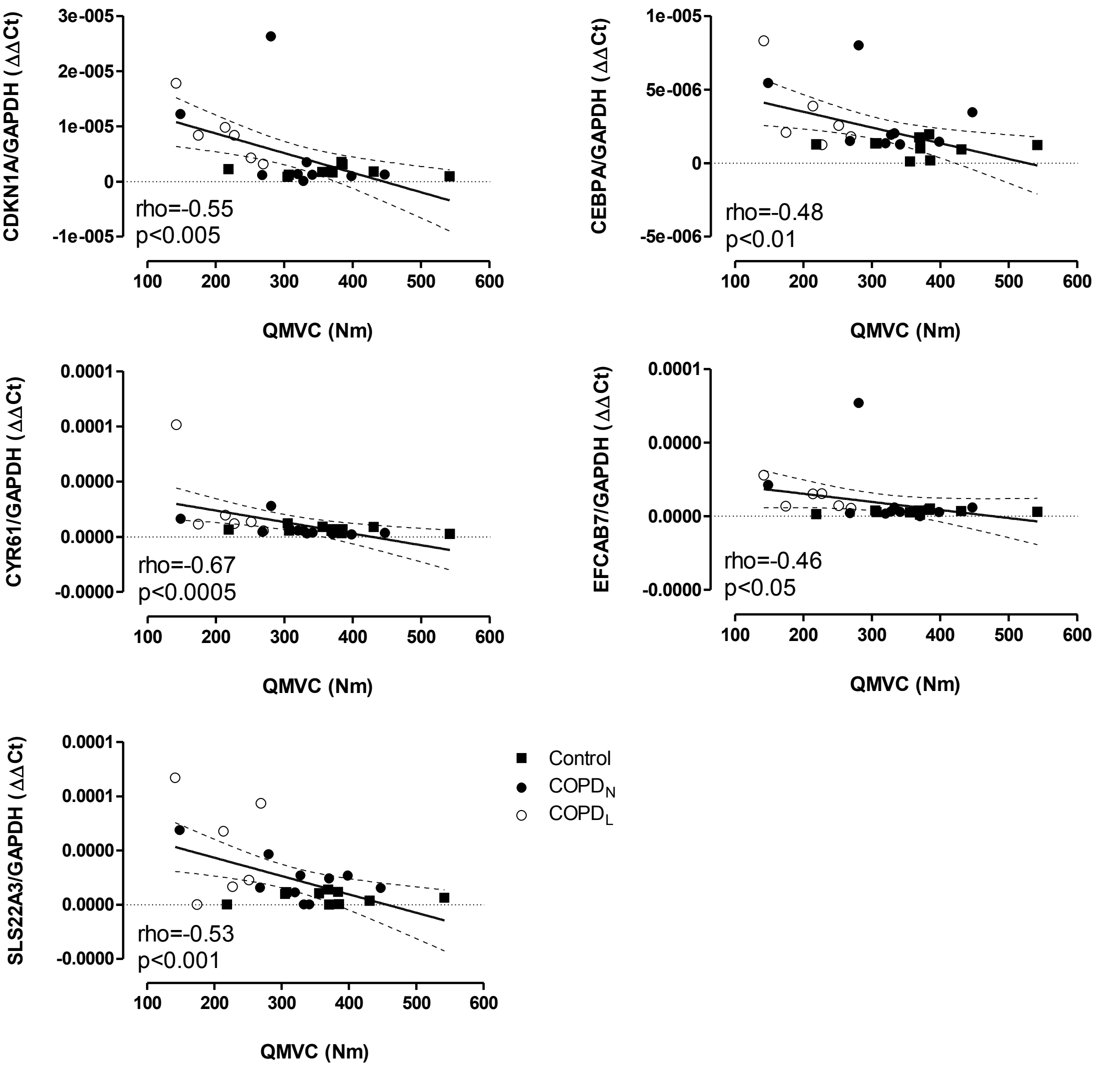

Figure S3: Correlations between QMVC (x axis) and qPCR gene expression corrected by GAPDH housekeeping gene (ΔΔCt) in COPD$_L$ (o), COPD$_N$ (●) and C (■) (y axis). Solid line represents regression line and dashed lines 95% CI.

REFERENCES

1. Rabinovich RD, E; Dunbar, D; Manning, J; Bastos, R; MacNee, W;: **Skeletal muscle gene expression in COPD patients with normal and low FFMI and healthy controls. A microarray gene expression analysis.** *Eur Respir J* 2009, **Suppl 34:**488s.
2. Rabinovich RD, E; Dunbar, D; Manning, J; Bastos, R; MacNee, W;: **Skeletal muscle gene expression in COPD patients with normal and low FFMI and healthy controls. A mycroarray gene expression analysis.** *Am J Respir Crit Care Med* 2010, **18:**A1553.
3. Miller MR, Hankinson J, Brusasco V, Burgos F, Casaburi R, Coates A, Crapo R, Enright P, van der Grinten CP, Gustafsson P, et al: **Standardisation of spirometry.** *Eur Respir J* 2005, **26:**319-338.
4. Schols AM, Broekhuizen R, Weling-Scheepers CA, Wouters EF: **Body composition and mortality in chronic obstructive pulmonary disease.** *AmJClinNutr* 2005, **82:**53-59.
5. **ATS statement: guidelines for the six-minute walk test.** *American Journal of Respiratory and Critical Care Medicine* 2002, **166:**111-117.
6. Spruit MA, Gosselink R, Troosters T, Kasran A, Gayan-Ramirez G, Bogaerts P, Bouillon R, Decramer M: **Muscle force during an acute exacerbation in hospitalised patients with COPD and its relationship with CXCL8 and IGF-I.** *Thorax* 2003, **58:**752-756.
7. Jones PW, Quirk FH, Baveystock CM, Littlejohns P: **A Self-complete Measure of Health Status for Chronic Airflow Limitation.** *American Review of Respiratory Disease* 1992, **145:**1321-1327.
8. Voorrips LE, Ravelli AC, Dongelmans PC, Deurenberg P, Van Staveren WA: **A physical activity questionnaire for the elderly.** *MedSciSports Exerc* 1991, **23:**974-979.
9. Garrod R, Bestall JC, Paul EA, Wedzicha JA, Jones PW: **Development and validation of a standardized measure of activity of daily living in patients with severe COPD: the London Chest Activity of Daily Living scale (LCADL).** *Respir Med* 2000, **94:**589-596.
10. Toth ZE, Mezey E: **Simultaneous visualization of multiple antigens with tyramide signal amplification using antibodies from the same species.** *The journal of histochemistry and cytochemistry : official journal of the Histochemistry Society* 2007, **55:**545-554.
11. Dow GS: **Effect of sample size and P-value filtering techniques on the detection of transcriptional changes induced in rat neuroblastoma (NG108) cells by mefloquine.** *MalarJ* 2003, **2:**4.
12. Team RDC: **A language and environment for statistical computing. R Foundation for Statistical Computing.** In *Book A language and environment for statistical computing. R Foundation for Statistical Computing* (Editor ed.^eds.). City; 2005.
13. Gentleman RC, Carey VJ, Bates DM, Bolstad B, Dettling M, Dudoit S, Ellis B, Gautier L, Ge Y, Gentry J, et al: **Bioconductor: open software**

development for computational biology and bioinformatics. *Genome Biol* 2004, **5:**R80.

14. Breitling R, Armengaud P, Amtmann A, Herzyk P: **Rank products: a simple, yet powerful, new method to detect differentially regulated genes in replicated microarray experiments.** *FEBS Lett* 2004, **573:**83-92.
15. Hong F, Breitling R, McEntee CW, Wittner BS, Nemhauser JL, Chory J: **RankProd: a bioconductor package for detecting differentially expressed genes in meta-analysis.** *Bioinformatics* 2006, **22:**2825-2827.
16. Hong F, Breitling R: **A comparison of meta-analysis methods for detecting differentially expressed genes in microarray experiments.** *Bioinformatics* 2008, **24:**374-382.
17. Dennis G, Jr., Sherman BT, Hosack DA, Yang J, Gao W, Lane HC, Lempicki RA: **DAVID: Database for Annotation, Visualization, and Integrated Discovery.** *Genome biology* 2003, **4:**P3.
18. Gallagher IJ, Scheele C, Keller P, Nielsen AR, Remenyi J, Fischer CP, Roder K, Babraj J, Wahlestedt C, Hutvagner G, et al: **Integration of microRNA changes in vivo identifies novel molecular features of muscle insulin resistance in type 2 diabetes.** *Genome medicine* 2010, **2:**9.
19. Calvano SE, Xiao W, Richards DR, Felciano RM, Baker HV, Cho RJ, Chen RO, Brownstein BH, Cobb JP, Tschoeke SK, et al: **A network-based analysis of systemic inflammation in humans.** *Nature* 2005, **437:**1032-1037.
