# Supplementary material for: Genome-wide mRNA expression profiling in vastus lateralis of COPD patients with low and normal fat free mass index and healthy controls
Source: Respir Res. 2015 Jan 8;16(1):1. doi: 10.1186/s12931-014-0139-5 (PMC4333166; doi:10.1186/s12931-014-0139-5)
Supplement: Additional file 2: Figure S1. — Lung Function and Smoking History. Lung Function and Smoking History in COPDL, COPDN and C. (*p < 0.05). [file 12931_2014_139_MOESM2_ESM.pdf]

Figure S1. Lung Function and Smoking History.

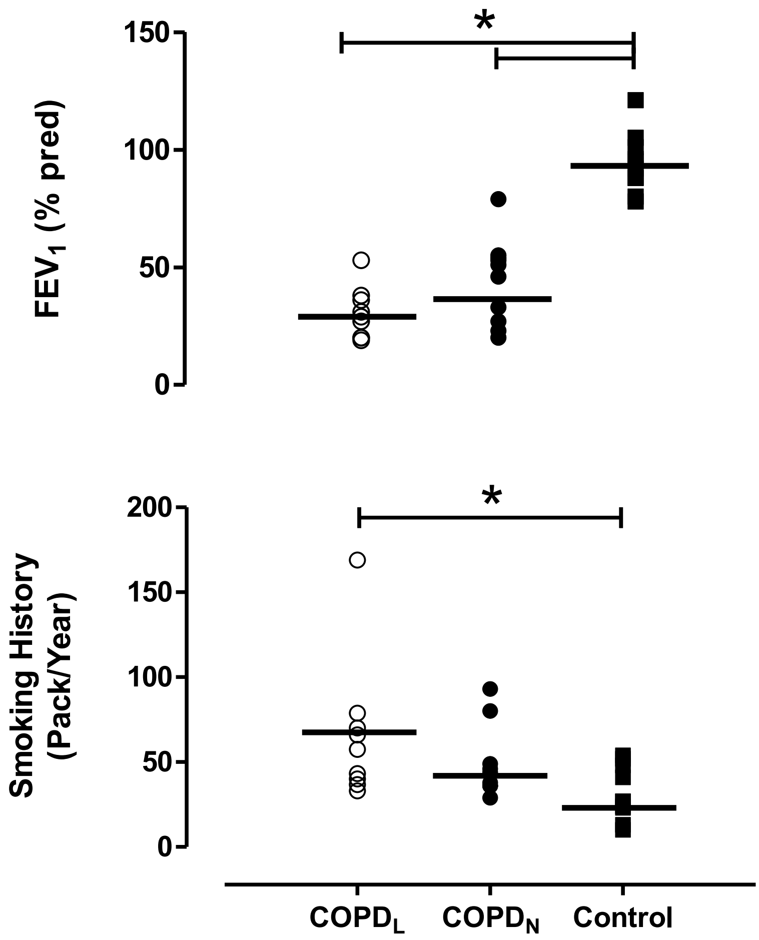

Figure S1: Lung Function and Smoking History in $COPD_L$, $COPD_N$ and C. (*p<0.05).
