# Supplementary material for: Genome-wide mRNA expression profiling in vastus lateralis of COPD patients with low and normal fat free mass index and healthy controls
Source: Respir Res. 2015 Jan 8;16(1):1. doi: 10.1186/s12931-014-0139-5 (PMC4333166; doi:10.1186/s12931-014-0139-5)
Supplement: Additional file 3: Table S1. — DEG between COPDL and both COPDN and C which varied with fibre type II area. List of up and down DEG genes between COPDL and both COPDN and C that varied with type II area in the whole populations. [file 12931_2014_139_MOESM3_ESM.pdf]

TABLE S1. DEG between $COPD_L$ and both $COPD_N$ and C which varied with fibre type II area.

Source	Probe	Gene Symbol	rho	p
Up-regulated genes	A_23_P100711	PMP22	-0.44	0.027
	A_23_P403445	CGREF1	-0.60	0.0013
	A_23_P146233	LPL	-0.42	0.032
Down-regulated genes	A_24_P335092	SAA1	0.49	0.012
	A_23_P13548	CHRLD	0.39	0.05
	A_23_P308763	FARP1	0.48	0.015
	A_24_P401294	FLJ35934	0.44	0.029

Table S1. *List of up and down DEG genes between $COPD_L$ and both $COPD_N$ and C that varied with type II area in the whole populations.*
