# Supplementary material for: Genome-wide mRNA expression profiling in vastus lateralis of COPD patients with low and normal fat free mass index and healthy controls
Source: Respir Res. 2015 Jan 8;16(1):1. doi: 10.1186/s12931-014-0139-5 (PMC4333166; doi:10.1186/s12931-014-0139-5)
Supplement: Additional file 4: Table S2. — DEG between COPDL and both COPDN and C which varied with fibre type II percentage. List of up and down DEG genes between COPDL and both COPDN and C that varied with type II fibre percentage in the whole populations. [file 12931_2014_139_MOESM4_ESM.pdf]

TABLE S2. DEG between $COPD_L$ and both $COPD_N$ and C which varied with fibre type II percentage.

Source	Probe	Gene Symbol	rho	p
Up-regulated genes	A_23_P59210	CDKN1A	0.39	<0.05
	A_23_P23221	GADD45A	0.47	<0.05
	A_23_P19733	SLS22A3	0.59	<0.005
	A_23_P34915	ATF3	0.43	<0.05
	A_23_P313482	ABRA	0.41	<0.05
	A_23_P161218	ANKRD1	0.38	<0.05
	A_24_P193295	RAB15	0.45	<0.05
Down-regulated genes	A_23_P57089	PMEPA1	-0.46	<0.05
	A_24_P319675	RAB10	-0.40	<0.05
	A_23_P146339	GPT	-0.46	<0.05
	A_24_P96961	SPSB1	-0.54	<0.01
	A_24_P368943	EVX1	-0.39	<0.05

Table S2. *List of up and down DEG genes between $COPD_L$ and both $COPD_N$ and C that varied with type II fibre percentage in the whole populations.*
