# Supplementary material for: Genome-wide mRNA expression profiling in vastus lateralis of COPD patients with low and normal fat free mass index and healthy controls
Source: Respir Res. 2015 Jan 8;16(1):1. doi: 10.1186/s12931-014-0139-5 (PMC4333166; doi:10.1186/s12931-014-0139-5)
Supplement: Additional file 5: Table S3. — DEG between COPDL and both COPDN and C which varied with fibre muscle function measured as QMVC. List of up and down DEG genes between COPDL and both COPDN and C that varied with QMVC in the whole populations. [file 12931_2014_139_MOESM5_ESM.pdf]

TABLE S3. DEG between $COPD_L$ and both $COPD_N$ and C which varied with fibre muscle function measured as QMVC.

Source	Probe	Gene	rho	p
Up-regulated genes	A_32_P234459	HLA-H	-0.46	<0.05
	A_23_P23221	GADD45A	-0.44	<0.05
	A_23_P22735	BEX2	-0.41	<0.05
	A_24_P193295	RAB15	-0.61	<0.005
	A_23_P403445	CGREF1	-0.39	<0.05
	A_23_P46426	CYR61	-0.58	<0.05
	A_24_P370946	CYR61	-0.60	<0.005
	A_23_P46429	CYR61	-0.59	<0.005
	A_24_P261734	SLC38A1	-0.44	<0.05
	A_23_P363399	SLC38A1	-0.45	<0.05
	A_23_P19733	SLC22A3	-0.41	<0.05
	A_23_P49338	TNFRSF12A	-0.50	<0.01
	A_23_P127584	NNMT	-0.39	<0.05
	A_32_P60459	OTUD1	-0.44	<0.05
	A_23_P34915	ATF3	-0.40	<0.05
	A_23_P161218	ANKRD1	-0.63	<0.0005
	A_32_P200144	IGHG1	-0.39	<0.05
Down-regulated genes	A_24_P413126	PMEPA1	0.48	<0.05
	A_23_P57089	PMEPA1	0.58	<0.005
	A_23_P146339	GPT	0.57	<0.005
	A_24_P96961	SPSB1	0.50	<0.01

Table S3. *List of up and down DEG genes between $COPD_L$ and both $COPD_N$ and C that varied with QMVC in the whole populations.*
