# Supplementary material for: Genome-wide mRNA expression profiling in vastus lateralis of COPD patients with low and normal fat free mass index and healthy controls
Source: Respir Res. 2015 Jan 8;16(1):1. doi: 10.1186/s12931-014-0139-5 (PMC4333166; doi:10.1186/s12931-014-0139-5)
Supplement: Additional file 6: Figure S2. — qPCR graphics for the validated genes between COPDL and both other groups COPDN and C. qPCR validated genes in COPDL, COPDN and C. (*p < 0.05). [file 12931_2014_139_MOESM6_ESM.pdf]

Figure S2. qPCR graphics for the validated genes between $COPD_L$ and both other groups $COPD_N$ and C.

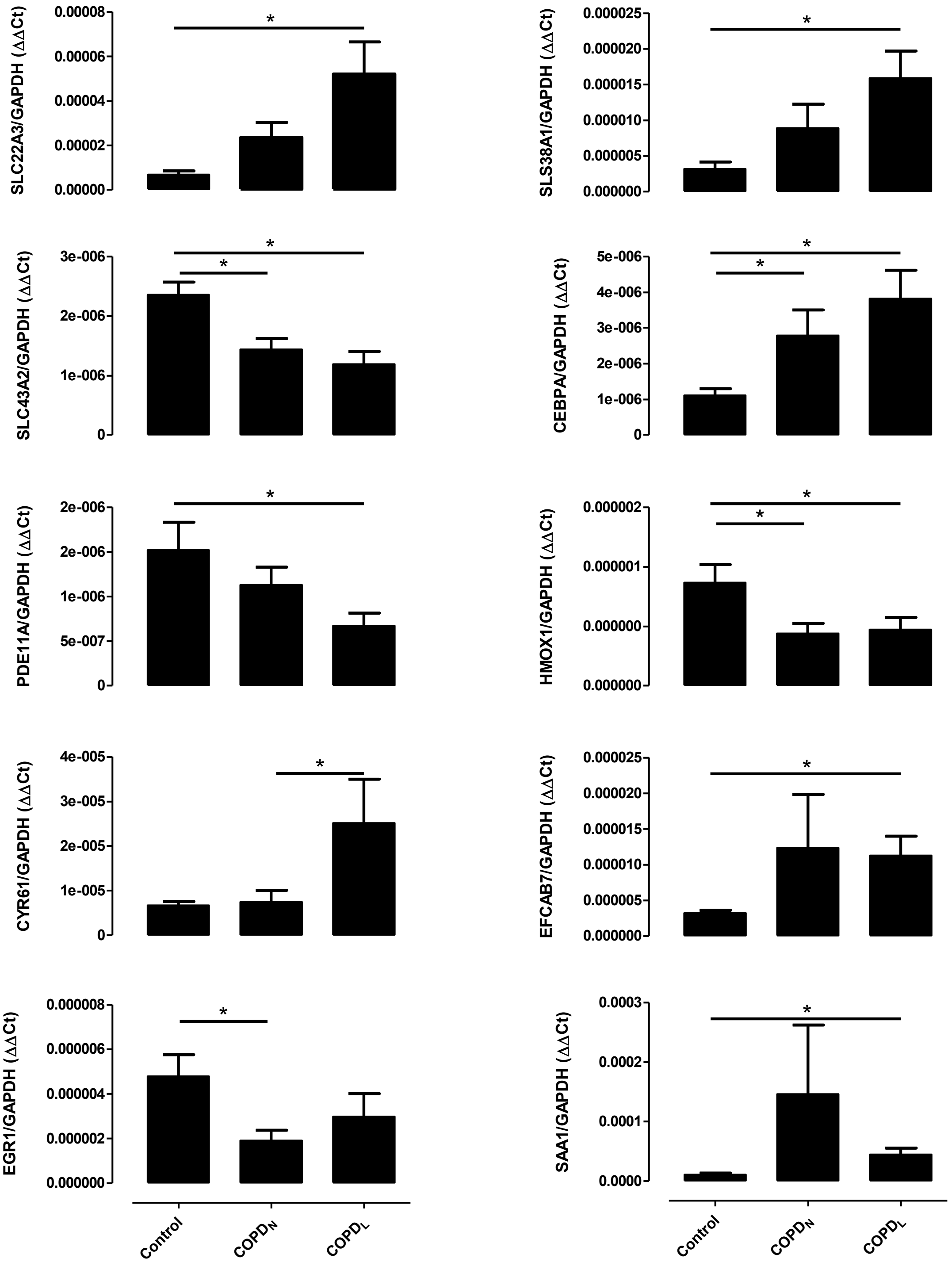

Figure S2: qPCR validated genes in $COPD_L$, $COPD_N$ and C. (*p<0.05).
