# Supplementary material for: Genome-wide mRNA expression profiling in vastus lateralis of COPD patients with low and normal fat free mass index and healthy controls
Source: Respir Res. 2015 Jan 8;16(1):1. doi: 10.1186/s12931-014-0139-5 (PMC4333166; doi:10.1186/s12931-014-0139-5)
Supplement: Additional file 7: Figure S3. — Correlations between qPCR genes and QMVC. Correlations between QMVC (x axis) and qPCR gene expression corrected by GAPDH housekeeping gene (ΔΔCt) in COPDL (o), COPDN (●) and C (■) (y axis). Solid line represents regression line and dashed lines 95% CI. [file 12931_2014_139_MOESM7_ESM.pdf]

Figure S3. Correlations between qPCR genes and QMVC

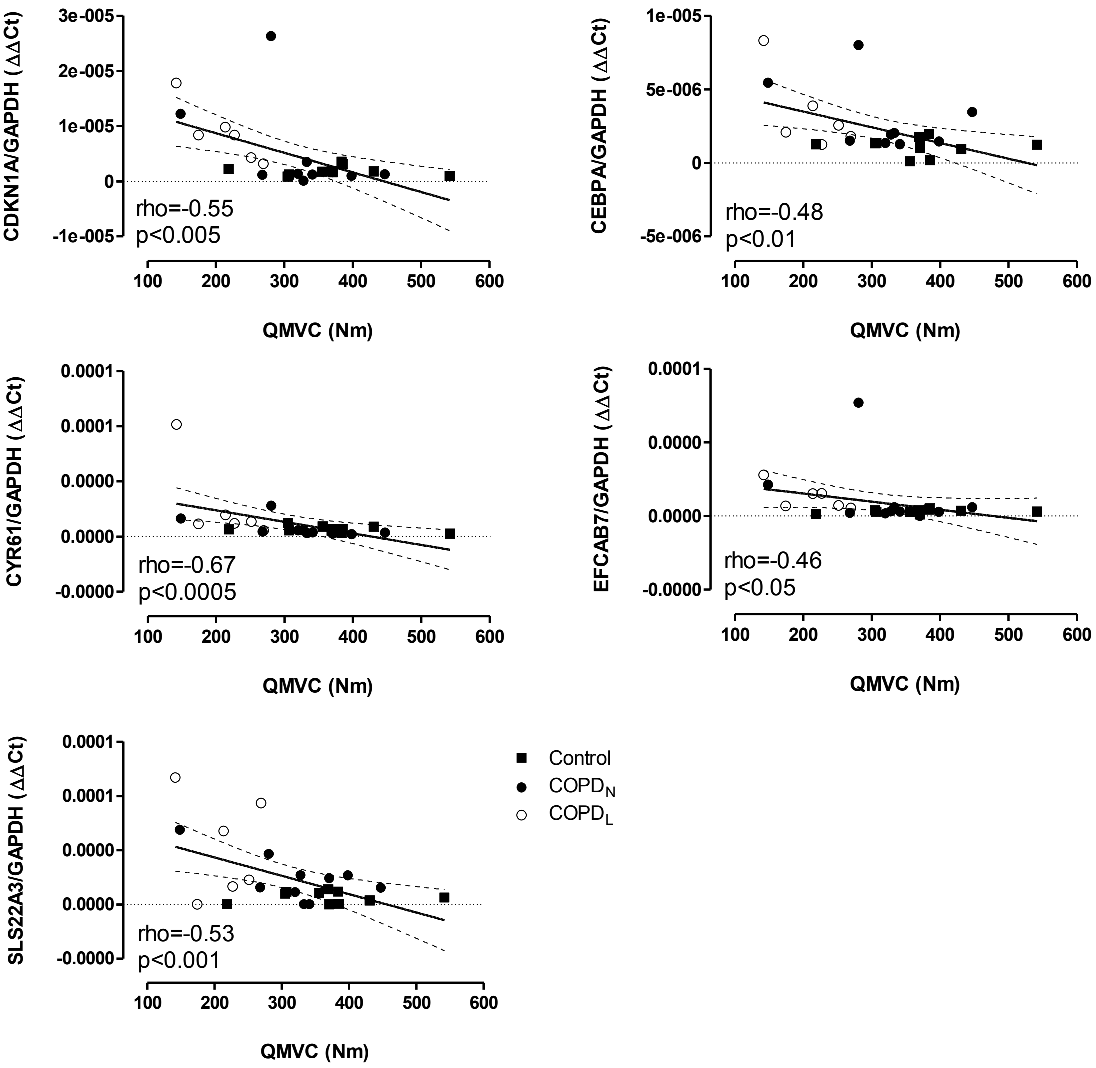

Figure S3: Correlations between QMVC (x axis) and qPCR gene expression corrected by GAPDH housekeeping gene (ΔΔCt) in COPD$_L$ (o), COPD$_N$ (●) and C (■) (y axis). Solid line represents regression line and dashed lines 95% CI.
